# Rigorously modeling self-stabilizing fault-tolerant circuits: An ultra-robust clocking scheme for systems-on-chip^☆^

**DOI:** 10.1016/j.jcss.2014.01.001

**Published:** 2014-06

**Authors:** Danny Dolev, Matthias Függer, Markus Posch, Ulrich Schmid, Andreas Steininger, Christoph Lenzen

**Affiliations:** a School of Engineering and Computer Science, The Hebrew University of Jerusalem, Edmond Safra Campus, 91904 Jerusalem, Israel; b Department of Computer Engineering, Vienna University of Technology, Treitlstrasse 3, 1040 Vienna, Austria; c Computer Science and Artificial Intelligence Laboratory, Massachusetts Institute of Technology, 32 Vassar Street, 02139 Cambridge, MA, USA

**Keywords:** Modeling framework, Clock synchronization, Hardware implementation, Experiments, Metastability, Dependability, Theoretical analysis, Hybrid state machines, Byzantine fault-tolerance, Self-stabilization

## Abstract

We present the first implementation of a distributed clock generation scheme for Systems-on-Chip that recovers from an unbounded number of arbitrary transient faults despite a large number of arbitrary permanent faults. We devise self-stabilizing hardware building blocks and a hybrid synchronous/asynchronous state machine enabling metastability-free transitions of the algorithm's states. We provide a comprehensive modeling approach that permits to prove, given correctness of the constructed low-level building blocks, the high-level properties of the synchronization algorithm (which have been established in a more abstract model). We believe this approach to be of interest in its own right, since this is the first technique permitting to mathematically verify, at manageable complexity, high-level properties of a fault-prone system in terms of its very basic components. We evaluate a prototype implementation, which has been designed in VHDL, using the Petrify tool in conjunction with some extensions, and synthesized for an Altera Cyclone FPGA.

## Highlights

• We introduce a novel modeling framework for fault-tolerant VLSI circuits.
• We cast a self-stabilizing clocking scheme from a companion article in this model.
• We discuss the implications of theory and model for the resulting implementation.
• We present the measures taken to avoid metastable upsets despite faults.
• We provide experimental data from a prototype FPGA implementation of the algorithm.

## 1 Introduction & related work

In the past, computers have essentially been viewed as monolithic, synchronous, fault-free systems. If at all, fault-tolerance has been introduced (i) to deal with limited, specific failures (e.g. errors in communication or data read from storage, which are usually handled via error-correcting codes), and (ii) at the level of distributed systems comprised of multiple machines that are fault-prone or subject to attacks (e.g. data centers or peer-to-peer applications, which use some form of replication). Except for critical systems and extreme operational conditions (e.g. medical or aerospace applications [1]), there has been little motivation to build systems that are robust on all levels from scratch, a process that involves redesigning—or even reinventing—the very basics of how computations are organized and performed.

Due to the tremendous advances of *Very Large Scale Integration* (VLSI) technology, this situation has changed. Enabled by ever decreasing feature sizes and supply voltages, modern circuits nowadays accommodate billions of transistors running at GHz speeds [2]. As a consequence, the assumption of chip-global (not to speak of system-global) synchrony [3] and no (or restricted) faults gradually became outdated [4]. Improved process technology and architectural-level fault-tolerance measures are common nowadays, and the lack of global synchrony has been tackled by accepting a certain level of asynchrony between different parts of the system.

In the most extreme form of this approach, computations are completely unsynchronized at all levels [5], which requires to synchronize all dependent activities (like sending and receiving of data) explicitly via handshaking. In contrast, *Globally Asynchronous Locally Synchronous (GALS)* systems [6] make use of local clock sources to drive synchronous computations within each *clock domain*. Note that, in the wider sense, most multiprocessors fall into this category, as there is usually no single common clock that drives all processors. GALS systems again can be divided into two general classes: One that operates asynchronously at the inter-domain level, and the other consisting of *multi-synchronous* systems [7,8] that provide some, albeit reduced, degree of synchronization among clock domains. The former class suffers from the drawback that, for inter-domain communication, either strong synchronizers or stoppable clocks must be foreseen [9]. After all, every bit of the sender's data must have stabilized at the receiver before the clock edge used for reading the data occurs. This is avoided in multi-synchronous systems, where high-speed inter-domain communication via FIFO buffers can be implemented due to the available global synchronization [10]. Since the latter abstraction is also very useful for other purposes, multi-synchronous GALS is preferable from the viewpoint of a system-level designer.

Naturally, establishing inter-domain synchronization comes at additional costs. While it is not too difficult to achieve and maintain in the absence of faults [11,12], the issue becomes highly challenging once faults of clocking system components enter the picture.

### 1.1 Contribution

We present an FPGA prototype implementation of a distributed clock generation scheme for SoC that self-stabilizes in the presence of up to f<n/3 faulty nodes. It incorporates the pulse algorithm from [13] that tolerates arbitrary clock drifts and allows for deterministic recovery and (re)joining in constant time if n−f nodes are synchronized; it stabilizes within time O(n) with probability $1-2^{-n}$ from any arbitrary state. An additional algorithmic layer that interacts weakly with the former provides bounded high-frequency clocks atop of it. Nodes executing the compound algorithm broadcast a mere constant number of bits in constant time. The formal proofs of the properties of the pulse synchronization algorithm and the derived high-frequency clocks are given in [13].

Deriving an implementation from the specification of the algorithm in [13] proved to be challenging, as the high-level theoretical model and formulation of the algorithm in [13] abstracts away many details. Firstly, it assumes a number of basic self-stabilizing modules above the level of gates and wires to be given. We devise and discuss self-stabilizing implementations of these building blocks meeting the specifications required by the high-level algorithm. Secondly, the algorithm's description is in terms of state machines performing transitions that are non-trivial in the sense that they do not consist of switching a single binary signal or memory bit. This requires careful consideration of metastability issues, since these state transitions are triggered by information from different clock domains. In order to resolve this issue, we introduce a generic *Hybrid State Transition Machine (HSTM)* that asynchronously starts a local synchronous execution of a state transition satisfying the model specification from [13]. Related to this matter, we thirdly discuss in detail how the algorithm and its implementation make a best effort to guard against metastable upsets. Here, we try to get the best out of the design decisions and rely on synchronizers only where absolutely necessary.

These non-trivial implementation issues and the complex interactions between the basic building blocks raise the question under which circumstances the high-level properties of the algorithm shown in [13] indeed hold for the presented implementation. To answer this question, we devised a model that is able to capture the behavior of the constructed modules, including faults, resilience to faults, and self-stabilization, in a hierarchical fashion. By specifying the desired behavior of modules in terms of the feasible output generated in response to their inputs, we can also *reason* about the behavior of (implementations of) modules in a hierarchical manner. This property is crucial, as it permits to determine conditions under which our implementation indeed satisfies the requirements by the abstract model used in [13], and then soundly conclude that if these conditions are met, all statements made in [13] apply to our implementation. Since our approach is highly generic and permits to adjust the granularity of the description in order to focus on specific aspects of the system, we believe it to be of general and independent interest in the context of devising fault-tolerant systems.

In order to verify the predictions from theory,^3^ we carried out several experiments incorporating drifting clocks, varying delays, and both transient and permanent faults. This necessitated the development of a testbed that can be efficiently controlled and set up for executing a large number of test runs quickly. In our 8-node prototype implementation, the compound algorithm generates 8-bit clocks that in all runs stabilized within $1.9\cdot 10^{6}d$ time (where *d* is the maximal end-to-end communication delay). In our testbed, which runs at roughly 100 kHz, this amounts to less than 12 s. For a system running at GHz speed, this translates to about a millisecond. We also observed that the deterministic stabilization mechanism designed for more benign conditions operates as expected, recovering nodes by about two orders of magnitude faster.

### 1.2 Organization of the article

In the next section, we summarize the obstacles and design goals that need to be considered for clock synchronization in our setting; we also introduce the basic building blocks assumed in [13], which perform typical operations used by fault-tolerant synchronization algorithms. Section 3 introduces the formal model, alongside illustrating examples and proofs of some basic properties. Subsequently, in Section 4 we cast the modules informally discussed earlier in our formal framework, and interpret nodes, protocols, and the synchronization problem as modules as well. In Section 5, we move on to the description of the algorithm from [13] in terms of this framework. We provide high-level intution on the purpose of its various components and summarize the main statements proved in [13]. Section 6 follows up with presenting our implementations of the basic modules specified in Section 2.2, including the HSTM. In this context, we will also cover our efforts to minimize the probability for metastable upsets. In Section 7 we describe the testbed setup, the experiments, and their results. Finally, in Section 8 we evaluate to what extent our design goals are met and give an outlook on future work.

## 2 On-chip clock synchronization

Our goal is to design a scalable hardware clock generation scheme that is resilient to arbitrary transient and permanent faults and carefully minimizes the risk of metastability. We will now discuss our objectives in more detail and explain why tackling them in conjunction proves to be much harder than achieving them individually.

In accordance with standard notions, in the following we will refer to clock domains as *nodes*, as they represent the smallest “independent” algorithmic building block we use. This is to be understood in the sense that we consider a node faulty if any one of its components is faulty, and non-faulty otherwise (irrespectively of whether other nodes behave correctly or not). Denoting by [i..j] the set {k∈N|i⩽k⩽j}, ultimately, each correct node i∈[1..n] must at all times *t* output a (discrete) *logical clock* $L_{i}(t)\in N$ that fulfills certain properties despite the aforementioned obstacles; most obviously, we strive for minimizing $\max_{i,j\in [1..n],t⩾0}\{L_{i}(t)-L_{j}(t)\}$.

### 2.1 Challenges

#### 2.1.1 Inexact local clocks and unknown message delays

When synchronizing clocks, one needs to face that clocks are not perfect and that it cannot be exactly determined how much time it takes to communicate a clock reading. These fundamental uncertainties entail that synchronization can never be perfectly accurate and must be an ongoing process [14]. We formalize these notions as follows.

Each node i∈[1..n] can make use of *local clocks* that are inexact and therefore drift (i.e., do not progress at the same rate). Since we are only concerned with synchronizing clock domains with each other, we do not care about Newtonian time. Instead, we describe the system in terms of a *reference time* satisfying that any correctly operating clock progresses at a speed between 1 and some constant *ϑ* with respect to the reference time t∈R. A (local) clock C:R→R that is *correct* during a period of reference time $[t^{-},t^{+}]\subseteq R$ guarantees that ∀*t*, $t^{\prime }\in [t^{-},t^{+}]$, $t<t^{\prime }$: $t^{\prime }-t⩽C(t^{\prime })-C(t)⩽\vartheta (t^{\prime }-t)$ (in particular, *C* is continuous and strictly increasing during $\left[t^{-},t^{+}\right]$).^4^ In contrast to many “traditional” synchronization settings, we would like to tolerate quite large relative clock drifts ϑ−1 of up to about 20%, as accurate and stable oscillators are not available in a System-on-Chip (SoC) at low costs. Tolerating such large drifts permits to utilize very simple ring oscillators even under heavily varying conditions (temperature, supply voltage, etc.) [15].

Node *i* communicates with node *j* via an *abstract* FIFO *channel* that (if correct) continuously makes *i*'s state available to *j*, albeit delayed by an unknown value between 0 and the *maximal delay d*. We denote the input port of the channel from node *i* to node *j* by $S_{i}$ and its output port by $S_{j,i}$. Node *i* also loops back its own state to itself on a channel. The time required for computations that are triggered by some communicated information is accounted for by *d* as well, i.e., *d* is an end-to-end delay.^5^ For the sake of a straightforward presentation, throughout this article we assume that all channels from node *i* to some node *j* are part of node *i*, i.e., faults of the channel are mapped to the sender node. We remark, however, that a more detailed treatment (as e.g. in [16]) can be beneficial and is supported by the modeling framework underlying this work.

#### 2.1.2 Transient faults

Increasing soft error rates of modern VLSI circuits [17], originating in ionizing radiation [18–21], cross-talk, and ground bouncing [22,23], make it vital to allow for recovery from transient faults. The most extreme transient fault scenario is that the entire system undergoes a period of an unbounded number of arbitrary faults.^6^ Algorithms that are capable of re-establishing regular operation after transient faults cease are called *self-stabilizing* [24]. This requirement is equivalent to stating that, if the system is fault-free, the algorithm converges to a valid state from an arbitrary initial configuration within a bounded time; we refer to this period as *stabilization time*. Due to this equivalency, self-stabilizing algorithms have the additional advantage of requiring no initialization, i.e., a self-stabilizing clocking system does not need to be booted with any initial synchrony.

For self-stabilizing algorithms, stabilization time is obviously an important quality measure. As the fundamental time unit of the system is *d*, i.e., the time span it takes to effectively communicate and process any piece of information with certainty, guarantees on the stabilization time are clearly always some multiple of *d*; the respective prefactor typically is a function of the number of nodes *n*, the number of sustainable or actual permanent faults, and the clock drift *ϑ*. In our context, the stabilization time is not only of relevance to whether waiting for stabilization is bearable in terms of the down-time of the system; it is important to understand that a failure of the synchronization layer will quickly result in incoherencies of operations on higher layers, entailing the threat of data loss or corruption, potentially without any possibility of future recovery.

Because of the need of maintaining accurate synchronization in the presence of drifting clocks, quite a few clock synchronization algorithms are self-stabilizing. In fact, conventional clock trees [3] are trivially self-stabilizing—after all, they simply disseminate the signal of a single oscillator throughout a chip. However, they cannot cope with any *permanent* fault of the clock source or the network distributing the clock. Similarly, one could easily make a system comprising several clock sources self-stabilizing, by picking one master clock and letting all other clocks synchronize to it. Again, this simplistic approach will fail if the master or its outgoing communication channels become faulty.

#### 2.1.3 Permanent faults

Sustaining functionality in the presence of permanent faults necessitates redundancy. More precisely, it is known that tolerating *f* worst-case faults (traditionally called Byzantine faults in this context) is impossible if n⩽3f (without cryptographic assumptions) [14,25].^7^ Hence, natural questions are whether assuming worst-case failures is too demanding and whether the fault model could be relaxed in order to circumvent the lower bound. Unfortunately, examining the lower bound reveals that it originates in the ability of a faulty node to communicate conflicting information to different receivers. This behavior can easily emerge from a faulty output stage in a circuit: If an analog voltage level in between the range for a valid “1” and that for a valid “0” is evaluated (for example due to a timing fault, a glitch on a signal line, or a defective driver output) by more than one receiver, some might read a “1” while others read a “0”. Note that this is a fundamental problem, as mapping the continuous range of possible voltages to discrete binary values entails that there is always a critical threshold close to which it is impossible to ensure that all receivers observe the same binary value. It is still an option to argue about the *spatial distribution* of (permanent) faults within the system, though, as we discuss in Section 8. However, in this article, we consider the worst case, which also motivates the choice of full connectivity^8^ between the nodes due to a respective impossibility result [26,27].

**Fig. 1:**
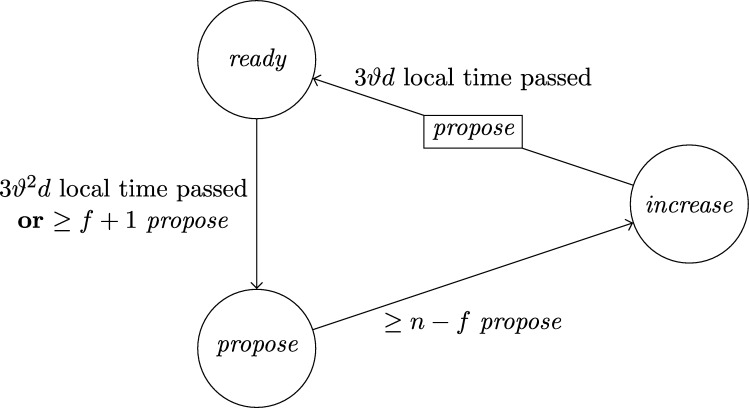
A simple synchronization algorithm tolerating *f* arbitrarily failing nodes granted that *n* ⩾ 3*f* + 1.

This lower bound entails that, due to their low connectivity, most existing distributed clock generation schemes [11,12,28,29] cannot cope with a reasonable number of worst-case faults. Nonetheless, dealing with up to *f* faults in a fully connected system of n⩾3f+1 nodes is—at least from a high-level perspective—still fairly easy, provided that we can rely on synchronization already being established. To illustrate this, consider the simple state machine of a node given in Fig. 1.

In the figure, the node's states are depicted in circles and the feasible state transitions are indicated by arrows. A node switches, for example, from state *ready* to state *propose* if the condition next to the arrow is satisfied. In this example, this means that either $3\vartheta^{2}d$ time has passed on its local clock since it switched to state *ready* or its incoming channels (including its loop-back channel) showed at least f+1 other nodes in state *propose* since it switched to state *ready*. This behavior is realized by each node i∈[1..n] having (binary) memory flags ${\mathrm{propose}}_{i,j}$ for each node j∈[1..n]: Node *i*'s flag ${\mathrm{propose}}_{i,j}$ is set to 1 at a time *t* iff $S_{i,j}(t)=\mathrm{propose}$ and the flag was in state 0 before. The flag is reset to 0 on node *i*'s state transition to *ready* (in the figure indicated by the rectangular box on the respective arrow). Deciding whether the transition condition is satisfied at time *t* thus boils down to checking whether the timeout condition is satisfied or at least f+1 of the *propose* memory flags are in state 1.

Now assume that each node runs a copy of this state machine, and at least n−f non-faulty nodes enter state *increase* during some time window [t,t+2d). As local clocks run at speeds between 1 and *ϑ*, all nodes will switch to state *ready* during [t+3d,t+2d+3ϑd). Hence, at the time when a node switches to *ready*, the delayed state information on the channels will not show non-faulty nodes in state *propose* any more. Therefore, no non-faulty node will switch to *propose* again due to memorizing f+1 nodes (at least one of which must be non-faulty) in state *propose* before the first non-faulty node switches to *propose*. Thus, the latter must happen because $3\vartheta^{2}d$ local time passed on a local clock, which takes at least until time t+3d+3ϑd>t+2d+3ϑd. By this time, all nodes will have switched to *ready*. This implies that at the time when the first node switches to *increase* again (which eventually happens because all n−f non-faulty nodes switch to *propose*), all nodes will already have switched to *ready*. Given that n⩾3f+1, we have that n−2f⩾f+1, i.e., if at *some* non-faulty node n−f channels show state *propose*, *any* node will observe f+1 channels in this state (though due to delayed communication maybe not at exactly the same instance in time). This implies that at most *d* time after the first node switched to *increase* again, all non-faulty nodes have switched to *propose*. Another *d* time later, all n−f non-faulty nodes will have become aware of this and have switched to *increase*, i.e., within a time window of 2*d*. Repeating this reasoning inductively and assuming that the nodes increase their logical clocks (that initially are 0) by 1 whenever they switch to *increase*, well-synchronized logical clocks are obtained: The maximum difference in time between any two correct nodes performing their *k*th clock tick, the *skew*, is at most 2*d* for the above algorithm.

A variation of this simple technique [30] is known for long and a closely related approach called DARTS has been implemented in hardware [31,32]. However, all these algorithms are not self-stabilizing. In fact, even if clocks would not drift, the delay *d* was arbitrarily small, and there was only a single faulty node (i.e., even if we allow for f>1, only one node is actually faulty), they still would not stabilize.

**Fig. 2:**
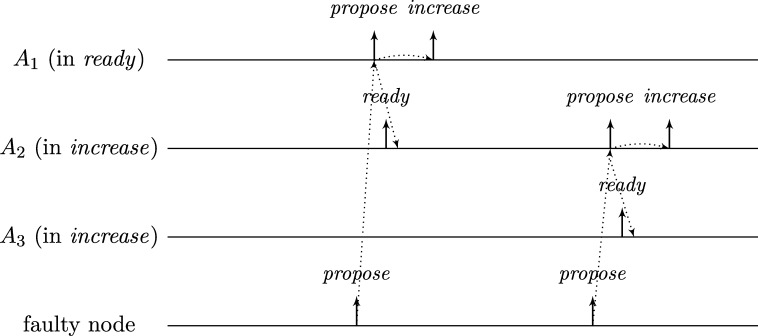
Part of a non-stabilizing execution of the algorithm shown in Fig. 1. Two iterations of the scheme described in the text are shown; after three are complete, the entire pattern is repeated.

To see this for the algorithm given in Fig. 1, first consider the following execution with n=3f+1, part of which is depicted in Fig. 2. The correct nodes are split evenly, into three subsets $A_{i}$, i∈{1,2,3}, of size *f*. Set $A_{1}$ initially is in state *ready*, with all memory flags corresponding to nodes in $A_{3}$ in state 1 and all other flags in state 0. The nodes in $A_{2}$ and $A_{3}$ are in state *increase*, with the timers of nodes in $A_{2}$ having progressed halfway towards expiring and the timers in $A_{3}$ just started (i.e., these nodes just left *propose*), and their *propose* signals are memorized by nodes in $A_{1}$. Just when the nodes in $A_{2}$ are about to switch to *ready*, the faulty node sends *propose* signals to the nodes in $A_{1}$, causing them to switch to *propose*. They will send *propose* signals, once receiving them memorize 2f+1=n−f nodes in state *propose*, and thus proceed to state *increase*. However, the nodes in $A_{2}$ will still observe the *propose* signals of the nodes in $A_{1}$ *after* resetting their memory flags upon switching to *ready*. Thus, we end up in the same situation, except that $A_{i}$ (indices modulo 3) takes the role of $A_{i-1}$. Repetition yields an execution that never stabilizes and has 3 sets of grossly desynchronized nodes that are not faulty. This execution can be generalized to n=kf+1 for integers k⩾3: we split the correct nodes in *k* sets of size *f* and make them proceed equidistantly spread in time through the cycle. The difference is that now more than one group will linger in states *ready* or *propose* upon arrival of the next; the crucial point is that the single faulty node retains control over when groups proceed to state *increase*. The cases n=kf+2 and n=kf+3 require more involved constructions; it should be intuitive, though, that with 2 actually failing nodes the above construction can be modified to operate with one or two of the sets containing f+1 nodes.

#### 2.1.4 Combining transient and permanent faults

Combining self-stabilization and resilience to permanent faults results in much more robust systems. Both properties synergize in that, as long as at all times there is some sufficiently large set (not necessarily the same!) of nodes that is non-faulty, an arbitrary number of transient faults is transparently masked, i.e., the system remains operational even though over time each individual component may repeatedly undergo transient failures and recover from them. This drastically increases the mean time until overall system failure: In a system that is not resilient to permanent faults, any fault will result in an immediate breakdown of guaranteed properties, whereas a system that is not self-stabilizing will fail (and might not recover without an external reboot) once the sum of faults exceeds one third of the nodes.^9^

There is a considerable body of work on distributed synchronization algorithms that are self-stabilizing as well as resilient to permanent faults. However, until recently, there has been no solution worth considering for hardware implementation. Known algorithms exhibit a prohibitively large communication complexity (i.e., nodes send Ω(n) bits over each channel in constant time) [33,34], incur an exponential stabilization time [35], require exponentially small clock drifts [36], or require much stronger assumptions on the system's behavior [37]. Recently, we proposed an approach that does not suffer from such drawbacks [13,38,39], whose implementation is the subject of this work.

#### 2.1.5 Metastability

In our specific setting, minimizing the potential for metastability is particularly demanding. Metastability results from violating a stateful circuit's input timing constraints, e.g., by changing the data input of a flip-flop at the time of the clock transition. While this can be safely avoided during normal operation, a faulty node might exhibit arbitrary timing and hence cause such a violation. As this can never be prevented in the first place if worst-case faults are considered, it is mandatory to guard the channels against propagating metastability, e.g. by using synchronizers. In order to minimize the required length of synchronizer chains, decreasing latency and area consumption (the latter also on higher layers of the system), however, it is beneficial to avoid the potential for upsets by construction wherever possible.

Apart from the (unavoidable) threat originating from faulty nodes, safely preventing timing violations is hindered by the lack of a common time base during the stabilization phase after an excessive number of transient faults. It has been shown that it is impossible to guarantee with certainty that no metastable upsets occur if the system is in an arbitrary initial state, even if all nodes adhere to the protocol [40]. Careful design is thus required in order to minimize the probability of upsets during stabilization, in particular since such upsets might obstruct the stabilization process.

Once the system stabilized, i.e., the non-faulty nodes are synchronized, the algorithm can use this synchronization to structure communication in a way that entirely avoids metastable upsets caused by non-faulty nodes. Thus, in the absence of faults, we require that the system operates metastability-free. Note that even this seemingly simple task is not trivial, as one cannot employ the classical wait-for-all paradigm: Doing so would imply that just a single non-responsive node would cause the entire system to deadlock. Therefore, when depending on other nodes in the decision to take a state transition, it is necessary to wait for at most n−f signals. Safely reading signals thus cannot rely on handshaking, but must be based on suitable monotonicity and/or timing conditions (guaranteed by the use of memory flags and local clocks, for example). The bounded-delay “interlocking condition” used in DARTS [32] and the simple algorithm in Fig. 1 are showcases for such techniques.

#### 2.1.6 Operating frequency vs. clock precision

In order to be practical, the logical clocks need to run at a frequency in the GHz range. While one could obviously utilize frequency multiplication to achieve this goal, this is not straightforward to build in the self-stabilizing context. After all, clock multipliers involve complex devices like phase-locked loops and are hence not obviously self-stabilizing. Moreover, for a fixed guaranteed skew (of say 2*d*), naive frequency amplification also increases the logical *clock imprecision* $\max_{i,j\in [1..n],t⩾0}\{L_{i}(t)-L_{j}(t)\}$ by the scaling factor, which may adversely affect certain services. For example, the size of the FIFO buffers used for inter-domain communication in [10] depends on the clock imprecision and must hence be adapted accordingly. On the other hand, by dividing frequencies, it is clearly possible to guarantee that $\max_{i,j\in [1..n],t⩾0}\{L_{i}(t)-L_{j}(t)\}=1$. Therefore, it is an important design goal to minimize clock imprecision while at the same time maximizing the frequency at which clocks run. Naturally, this becomes much more involved due to the design goals already presented.

#### 2.1.7 Scalability

Being able to meet all the above design goals is meaningless if one cannot control the amount of resources devoted to the task of clock generation. Pivotal issues are the following:

• **Area consumption:** The chip area used by the components of the synchronization algorithm decomposes into the area consumed by the nodes and their interconnections. The former can be captured by the *gate complexity*, i.e., the number of (constant fan-in) gates required to perform the algorithm's computations. The latter significantly depends on the chip layout, which is highly application-dependent and hence outside our scope of control. It is clear, however, that the number of channels and their bandwidth play a crucial role.
• **Communication complexity:** Apart from whether two nodes are connected or not, it is of interest how many wires are required. This is well-represented by the *bit complexity* of an algorithm, i.e., the number of bits it exchanges per time unit between communication partners. Note that while the number of wires can be reduced by means of time division, this will require additional memory and computational resources on the receiver's side and increase the communication delay. In any case, it is highly desirable to devise algorithms of (small) constant bit complexity. Moreover, broadcasting the same information to all nodes instead of different information to different receivers is to be preferred, as it allows us to use communication buses.
• **Stabilization time:** For reasons stated earlier, we would like to minimize the stabilization time. In particular, it is not good enough to know that an algorithm eventually stabilizes, as the required time might be well above what makes the algorithm self-stabilizing in any practical sense.
• **Resilience:** The number *f* of faults that can be concurrently sustained without losing synchronization or the capability to stabilize should grow with system size, as otherwise a larger system will suffer from more frequent outages. Note that while we must accept that stabilization is a random process (due to the unavoidable probability of metastable upsets), we demand that a system that is stable will always remain so as long as there are not too many faults (including upsets). As mentioned earlier, a lower bound shows that always f<n/3, giving a precise meaning to “too many” here.
• **Delays:** As the maximal delay *d* accounts both for the delay incurred by communication as well as computation, it is vital to minimize both. Notwithstanding the fact that the communication delay and computing speed is mostly determined by parameters outside our control (technology, spatial distances, number of nodes, etc.), minimizing the gate complexity and, in particular, the depth of the circuits implementing the nodes' algorithms (that determine the computing delays) is important.
• **Metastability:** In larger and faster systems, the number of events per time unit that could cause metastable upsets is obviously larger. Therefore, it is vital to safely exclude metastability from occurring during regular operation by construction.^10^ We admit metastability only during rare exceptional phases of system operation where it cannot be avoided in principle, like during stabilization or in case of faults. As the probabilities for metastable upsets are hard to quantify even in a final product, we do not use a “hard” measure here.^11^
• **Connectivity:** In order to facilitate efficient placement and routing on a chip, it is vital to ensure that the communication network is sparse. Also, a sparse network will consume less area and is beneficial to fault containment.^12^ Tackling this issue is subject to our future work and hence beyond the scope of this article, however.
• **Clock size:** If the logical clocks have too few bits, i.e., overflow too frequently, they might be unsuitable for the application logic of the SoC. The algorithm we present in this article can in principle provide clocks of arbitrary bounded size. However, its stabilization time would grow linearly with the maximum clock value once we scale above 8-bit clocks. In a recent publication, we show how to construct larger clocks efficiently [41].

### 2.2 Typical modules for clock synchronization protocols

We next introduce the basic modules that are assumed by the model used in [13]. We first give an intuitive description of the required modules. Subsequently, we introduce a novel formal framework for specifying self-stabilizing fault-tolerant modules and specify our basic modules in this framework. Any implementation satisfying this specification can be plugged into the high-level algorithm in order to yield a system guaranteeing the properties proved in [13].

We now list the building blocks beyond standard logic gates that will explicitly or implicitly be used by the algorithm presented in Section 5. Each of these building blocks computes output signals that are constrained by (the history of) its input signals. If the logic function implies an output transition in reaction to an input change, this transition is not required to occur immediately; it must occur within a known time bound, however. Given the time bounds for the individual modules and the connecting wires, one can compute the maximum delay *d*. Moreover, informally speaking, it must be avoided that a single change in the input(s) causes multiple transitions of the output signal, as this could undermine the high-level algorithm's logic. Note also that statefulness, i.e., any sort of memory (including positive feedback loops), bears the potential for metastable upsets and requires careful attention in order to ensure self-stabilization.^13^ Purely combinational elements, on the other hand, differ in their ability to prevent metastable inputs from reaching the output under certain conditions.

Each node will be a union of state machines that communicate via channels (both among each other and with remote nodes) and are composed of standard logic gates and all other modules we describe below. Fig. 5 depicts such a state machine.

• **Communication channels.** We previously introduced the communication channels from node *i* to node *j* as abstract devices that convey the states with a delay of at most *d* that also accounts for computations. Viewed as a module, the (physical) communication channels do account for the time to communicate the state information only, whereas computations are performed by standard logic gates and the modules we will describe next. A communication channel of this type simply maps its input signal to its output signal. The reason why communication channels are nonetheless listed as modules here is that encoding a non-binary state signal in a glitch- and metastability-free manner is a non-trivial task, as in the absence of (reliable) synchrony both parallel and sequential communication present challenges. In our abstraction, this encoding is performed by the channel, which requires additional logic and thus potentially results in delays beyond the mere wire delays as well as the necessity to consider issues concerning metastability and self-stabilization.
• **Memory flags.** These are just simple binary storage elements that can be *set* to 1 by means of one input signal and can be *reset* to 0 by a second input signal; their state is externally accessible via an output signal. Simply put, a memory flag just “remembers” which input signal was 1 most recently. In our algorithms, memory flags will be used to memorize wether an input signal from a remote node was in state 1 at some time after the most recent reset upon a state transition of one of the node's state machines (in Fig. 1, e.g., a node resets its *propose* flags when switching from *increase* to *ready*).
• **Threshold gates.** Frequently, nodes will need to decide whether a certain threshold number (f+1 or n−f) of signals (or sets of signals) satisfy some Boolean predicate (e.g., the conditions for switching to *propose* and *increase* in Fig. 1 involve such a threshold). A threshold gate takes the respective binary input signals and outputs 1 if the threshold is reached and 0 otherwise.
• **Watchdog timers.** Watchdog timers are the nodes' sense for the progress of time. Each timer (T,s,C), where *T* in $R^{+}$ is a duration and *C* a clock, is associated with state *s* and is either *expired* (output 1) or *not expired* (output 0). The timer is *reset* to 0 when the node's state switches to *s* and will *expire* after *T* time has passed according to clock *C*, unless it is reset again beforehand. Hence, if it is reset at (reference) time *t*, it will expire at some time $t^{\prime }\in [t+T/\vartheta ,t+T]$. For instance, the transition to *ready* in Fig. 1 is triggered by a watchdog timer.
• **Randomized watchdog timers.** A randomized watchdog timer is identical to a regular watchdog timer except that the duration *T* of the timeout is drawn from a (bounded) random distribution D over $R^{+}$. That is, if randomized timer (D,s,C) is reset at time *t*, it will expire at time $t^{\prime }\in [t+T/\vartheta ,t+T]$, where *T* is drawn from D. How this randomness is implemented subtly affects resilience and security properties of the system, see [13] for a formal definition of the way randomness is to be employed. For the purpose of this article, we confine ourselves to pointing out that, essentially, we require that faulty nodes do not have access to the value *T* drawn from D before time $t^{\prime }$.
• **State transition modules.** The algorithm does not demand zero-time state transitions. Nevertheless, it is non-trivial to ensure metastability-free state transitions and consistent memory states in our setting. On a transition from state *s* to state $s^{\prime }$, the node needs to switch its state signal (i.e., the input to its outgoing communication channels) exactly once from *s* to $s^{\prime }$ and reset any memory flag that is to be reset upon transitioning from *s* to $s^{\prime }$. This is complicated because the condition under which the state transition occurs, a Boolean predicate over a blend of signals from incoming communication channels and local modules' outputs, may be satisfied for a brief period of time only, or a condition for switching to a different state $s^{\prime \prime }$ might become satisfied at almost the same instant. Even worse, it may e.g. be required that memory flags whose output is part of a predicate expressing the condition for switching from *s* to $s^{\prime }$ are to be reset upon the transition to $s^{\prime }$. Resolving these issues is the purpose of a state transition module that controls the safe transition from one state to another.

## 3 A formal modeling framework for self-stabilizing fault-tolerant circuits

In this section, we introduce a novel formal framework for specifying self-stabilizing fault-tolerant modules. It is a non-trivial extension of [32,42] that allows us to rigorously express the properties related to self-stabilization as used in [13]. Using this framework, we then give a precise formal specification of our basic modules' behavior.

### 3.1 Signals

We define (the trace of) a *signal* to be a timed event trace over a finite alphabet S of possible signal states: Formally, signal σ⊆S×R. The elements of *σ* are called *events*, and for each event (s,t) we call *s* the *state of event* (s,t) and *t* the *time of event* (s,t). Note that we allow for events (s,t) and $(s,t^{\prime })\in \sigma$, where $t<t^{\prime }$, without having an event $(s^{\prime },t^{\prime \prime })\in \sigma$ with $s^{\prime }\neq s$ and $t<t^{\prime \prime }<t^{\prime }$. In this case, we call event $\left(s,t^{\prime }\right)$ *idempotent*. In general, a signal *σ* is required to fulfill the following conditions:

(i) From (s,t)∈σ and $(s^{\prime },t)\in \sigma$ follows that $s=s^{\prime }$.
(ii) For each time interval $[t^{-},t^{+}]\subseteq R$ of finite length, the number of non-idempotent events in *σ* with times within $\left[t^{-},t^{+}\right]$ is finite.
(iii) For any time *t*, there exists an event $(s,t^{\prime })\in \sigma$ with $t^{\prime }⩽t$.

We say that *signal σ switches to s* at time *t* iff event (s,t)∈σ is not idempotent. Due to property (ii), there is always a non-zero amount of time between two such events. These events describe when the corresponding physical signal undergoes an actual transition. Therefore, we define that signals *σ* and $\sigma^{\prime }$ are *equivalent*, iff they differ in idempotent events only, and identify all signals of an equivalence class. Each equivalence class [σ] contains a unique signal $\sigma_{\max}$ that contains an event for each time t∈R. We identify this signal (and thus the entire class) with the function that maps each time *t* to the state σ(t):=s satisfying that $(s,t)\in \sigma_{\max}$. We call σ(⋅) the *state function* of signal *σ*, and σ(t) the *state of signal σ at time t*. Note that since the state function of a signal *σ* depends on [σ] only, we may add or remove idempotent events at will without changing the state function.

### 3.2 Modules and executions

Each module comprises a (possibly empty) set of *input ports* and a (possibly empty) set of *output ports*. These sets must be disjoint, i.e., we do not allow a module's output to be identical to one of its inputs; it may be identical to the input port of another module, however. An *execution of ports P on interval* I⊆R assigns a state to each port in *P* at any time in *I*. For convenience of notation, for any port *p*, we identify *p* and its state function whenever the respective execution is clear from the context. Moreover, we may omit the “on R” for all executions for which I=R when clear from the context. An *execution of module M on I* is an execution of *M*'s input and output ports on *I*. The *restriction of execution* E on *I* to $I^{\prime }\subseteq I$ is the restriction of all of E's state functions to the interval $I^{\prime }$. Let E be an execution of ports *P* on *I*. Then the *restriction of execution* E on *I* to ports $P^{\prime }\subseteq P$ is the execution of ports $P^{\prime }$ on *I* with state functions equal to E's state functions on $P^{\prime }$.

Besides input and output ports, a module *M* further has a *module specification*. We allow two kinds of module specifications for a module *M*, distinguishing between *basic* and *compound* modules.

##### Basic modules.

In this case, the module specification is a function $\Phi_{M}$ that, for all *I*, maps *every* execution of the module's input ports on *I* to a *set* of executions of the module's output ports on *I*. The intended meaning of $\Phi_{M}$ is to map each and every conceivable execution of *M*'s input ports on *I* to the resulting possible reactions of *M* during the same time, which may be many different ones. For example, a module that may behave arbitrarily for some input execution $E_{\text{in}}$ is specified by setting $\Phi_{M}(E_{\text{in}})$ to be the set of *all* conceivable output executions. For a basic module *M* we say that execution E of *M* on some *I* is *feasible* iff $E_{\text{out}}\in \Phi_{M}(E_{\text{in}})$, where $E_{\text{in}}$ and $E_{\text{out}}$ are the restrictions of execution E to *M*'s input and output ports, respectively.

We require two properties for $\Phi_{M}$ to hold:

(i) *Non-emptiness*: $\Phi (E_{\text{in}})\neq \emptyset$ for all executions $E_{\text{in}}$ of *M*'s input ports.
(ii) *Properness*: Any restriction (in time) of a feasible execution of *M* is feasible.

These properties are motivated by the facts that (i) any given input of a correctly operating module will produce some output and (ii) correct operation on a given time interval implies correct operation on any subinterval of this interval.

Example 3.1 A simple basic module is a (zero-time) *inverter* with (binary) input port *i*, (binary) output port *o*, and module specification $\Phi_{\mathrm{Inv}}$ defined by: For each interval I⊆R and each execution $E_{\text{in}}$ of input port *i*, an execution $E_{\text{out}}$ of output port *o* on *I* is in $\Phi_{\mathrm{Inv}}(E_{\text{in}})$ iff for all t∈I it holds that o(t)=¬i(t).

Example 3.2 As an example of a timed basic module, consider a *fixed-delay channel* with input port *i*, output port *o*, and delay d>0. Its module specification $\Phi_{C}$ is defined by: For each interval $[t^{-},t^{+})\subseteq R$ and each execution $E_{\text{in}}$ of input port *i* on $\left[t^{-},t^{+}\right)$, an execution $E_{\text{out}}$ of output port *o* on *I* is in $\Phi_{C}(E_{\text{in}})$ iff for all $t\in [t^{-}+d,t^{+})$ we have that o(t)=i(t−d).

Clearly, a basic module needs to adhere to $\Phi_{M}$ only on intervals *I* during which it is correct, and may behave arbitrarily when it is faulty. Subtle issues originate in the fact that a module may become correct after an earlier (transient) fault, in the sense that its internal components work as intended afterwards. At this point in time, it may or may not be the case that all traces of the transient fault have been vanished from the internal state of the module.

The typical use of basic modules is the description of a (sub)problem. For instance, the module specification of a threshold module will be such that the output is required to indicate whether a certain number of binary input ports is in state 1. Basic modules are then employed with the understanding that they require an implementation matching their specification.

This use of basic modules in our algorithms entails that correct modules have correct internal states. Although a basic module description abstracts away its internal state, this property can be characterized in a natural way by another constraint on the module specification: We say that the specification of some module *M* is *extendable* iff each feasible execution of *M* on some interval $[t^{-},t^{+})\subset R$ is the restriction of a feasible execution of *M* on R. In other words, for each execution on some interval $\left[t^{-},t^{+}\right)$, (i) a fault-free history exists that leads to the internal state of the module at time $t^{-}$ (and therefore, for the given input signals, to the same execution on $\left[t^{-},t^{+}\right)$), and (ii) the module is capable of continuing to operate correctly in the future (i.e., there is no fault-free execution that eventually cannot be continued in a way that adheres to $\Phi_{M}$). While (ii) should always be true for essentially the same reason that we demand non-emptiness for, (i) can be seen as the requirement of a correct internal state: For a correct module with an extensible specification, it is required that both all internal components of a physical implementation of the module operate according to their specification and all traces of an earlier transient fault (if any) from the internal state must have worn off, in the sense that it could have been reached by a non-faulty history. Most of the basic module specifications we are going to introduce will be extendable. However, there are also basic modules with non-extendable specifications; we will provide an example later on.

The next lemma shows that any extendable module specification is uniquely characterized by its values on input executions of the respective module on R.

Lemma 3.3 *Any function Φ that maps each execution* $E_{\text{in}}$ *of a set of input ports* $P_{\mathrm{in}}$ *on* R *to a non-empty set of executions* $E_{\text{out}}$ *of a set of output ports* $P_{\mathrm{out}}$ *on* R *specifies a unique module M such that* (i) $\Phi_{M}$ *is extendable and* (ii) $\Phi_{M}(E_{\text{in}})=\Phi (E_{\text{in}})$ *for each execution of M's input ports* $P_{\mathrm{in}}$ *on* R*. Conversely, for each module M whose specification is extendable,* $\Phi_{M}$ *is uniquely characterized by its values on executions of ports* $P_{\mathrm{in}}$ *on* R*.*

Proof We show first that for a function *Φ* as assumed by the lemma there exists a module *M* such that $\Phi_{M}(E_{\text{in}})=\Phi (E_{\text{in}})$ for executions $E_{\text{in}}$ of ports $P_{\mathrm{in}}$ on R and $\Phi_{M}$ is extendable. To this end, we simply define that *M* is a module with input ports $P_{\mathrm{in}}$ and output ports $P_{\mathrm{out}}$ whose specification $\Phi_{M}$ is given by $E_{\text{out}}\in \Phi_{M}(E_{\text{in}})$ iff $E_{\text{in}}$ and $E_{\text{out}}$ are restrictions of executions $E_{\text{in}}^{\prime }$ and $E_{\text{out}}^{\prime }$ on R, respectively, such that $E_{\text{out}}^{\prime }\in \Phi (E_{\text{in}}^{\prime })$. Clearly, $\Phi_{M}$ satisfies properness and extendability by construction. Regarding non-emptiness, we can extend any input execution $E_{\text{in}}$ on some interval *I* arbitrarily to an execution $E_{\text{in}}^{\prime }$ on R. By assumption, $\Phi (E_{\text{in}}^{\prime })\neq \emptyset$, so there must be some execution $E_{\text{out}}^{\prime }\in \Phi (E_{\text{in}}^{\prime })$. By definition, its restriction $E_{\text{out}}$ to *I* is in $\Phi_{M}(E_{\text{in}})$, proving non-emptiness. Hence $\Phi_{M}$ is a valid specification of a module *M* with input ports $P_{\mathrm{in}}$ and output ports $P_{\mathrm{out}}$.

We now establish the second claim of the lemma, which will also show that the module *M* we constructed from *Φ* is unique and thus complete the proof. To this end, let $E_{\text{in}}$ and $E_{\text{out}}$ be any executions of the input and output ports of *M*, respectively. If $E_{\text{out}}\in \Phi_{M}(E_{\text{in}})$, by extendability $E_{\text{in}}$ and $E_{\text{out}}$ are restrictions of executions $E_{\text{in}}^{\prime }$ and $E_{\text{out}}^{\prime }$ on R, respectively, such that $E_{\text{out}}^{\prime }\in \Phi_{M}(E_{\text{in}}^{\prime })$. On the other hand, if $E_{\text{out}}\notin \Phi_{M}(E_{\text{in}})$, properness necessitates that there are no such executions $E_{\text{in}}^{\prime }$ and $E_{\text{out}}^{\prime }$. This shows the second statement and the lemma follows.  □

Enabled by this lemma, we will specify most of our basic modules by defining $\Phi_{M}(E_{\text{in}})\neq \emptyset$ for input executions $E_{\text{in}}$ on R only. For instance, the fixed delay channel from Example 3.2 can now be specified equivalently as follows.

Example 3.4 A fixed-delay channel with input port *i*, output port *o*, and delay d>0 is a basic module with extendable module specification $\Phi_{C}$ defined by: For each execution $E_{\text{in}}$ of input port *i* on R, an execution $E_{\text{out}}$ of output port *o* on R is in $\Phi_{C}(E_{\text{in}})$ iff for all t∈R we have that o(t)=i(t−d).

Because we do not need to describe *partial* feasible executions on a finite interval $I=[t^{-},t^{+})$ here, the fact that the condition o(t)=i(t−d) applies only to a subinterval of *I* in Example 3.2 becomes superfluous: (−∞+d;∞)=R, so no additional specification is required to describe the module's behavior near the lower bound of intervals $\left[t^{-},t^{+}\right)$ with $t^{-}\neq \infty$. By contrast, in Example 3.2, we had to specify this explicitly, by stating that *any* behavior of the output in $\left[t^{-},t^{-}+d\right)$ is feasible (by leaving the behavior unspecified in this interval).

Whereas the specification in Example 3.4 simplifies the one of Example 3.2 only marginally, this is very different for the modules exhibiting more complex relations between input and output introduced later on: Accurately describing all suffixes of a non-trivial partial execution can be error-prone and may result in unnecessarily involved module specifications.

##### Compound modules.

In this case, the module *M* is given by a finite set $S_{M}$ of *submodules* of *M*. These submodules are interconnected, i.e., an output port of one submodule can be the input port of another, and the ports of *M* may also be ports of submodules. We require that $S_{M}$ is *well-formed*, i.e., the following conditions are satisfied:

1. For each output port *p* of *M*, there is exactly one submodule $S\in S_{M}$ such that *S* has output port *p*.
2. For each input port *p* of a submodule of *M*, either *p* is an input port of *M*, or there is exactly one submodule $S\in S_{M}$ such that *S* has output port *p*.

The idea behind this definition is to “plug together” ports in a way such that no port is “driven” by more than one port or be left “floating”.

For a compound module *M*, we now define the *corresponding basic module* β(M). β(M)'s input and output ports are the input and output ports of *M*, whereas its module specification $\Phi_{\beta (M)}$ is defined as follows: Let E be an execution of *M* on I⊆R and denote by $E_{\text{in}}$ and $E_{\text{out}}$ its restrictions to *M*'s input and output ports, respectively. Then $E_{\text{out}}\in \Phi_{\beta (M)}(E_{\text{in}})$, iff there exists an execution $E_{M,S_{M}}$ on *I* of all ports of modules in $\{M\}\cup S_{M}$, such that, for each module $S\in S_{M}$, the restriction of execution $E_{M,S_{M}}$ to the ports of *S* is feasible for *S*, and $E_{M,S_{M}}$ restricted to *M*'s ports is equal to E. Note that $\Phi_{\beta (M)}$ satisfies properness: This follows directly from the definition and properness of all submodules if all submodules are basic, and for more complex modules by structural induction. We further require that the choice of $S_{M}$ is such that $\Phi_{\beta (M)}$ fulfills non-emptiness. Hence, β(M) is indeed a basic module with specification $\Phi_{\beta (M)}$. We say that execution E of *M* is *feasible for M* iff it is feasible for the corresponding basic module β(M). Intuitively, this means that we consider a compound module correct whenever all its submodules are correct.

**Fig. 3:**
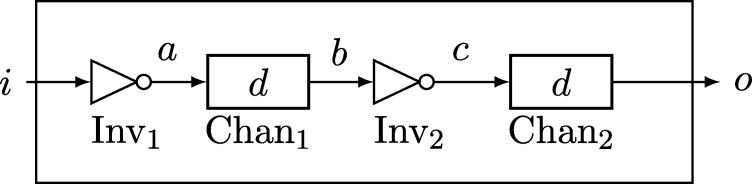
Compound module InvChain.

Example 3.5 Consider a compound module InvChain with (binary) input port *i* and (binary) output port *o*. InvChain is specified by the set of modules $S_{\mathrm{InvChain}}=\{{\mathrm{Inv}}_{1},{\mathrm{Inv}}_{2},{\mathrm{Chan}}_{1},{\mathrm{Chan}}_{2}\}$, where ${\mathrm{Inv}}_{1}$ and ${\mathrm{Inv}}_{2}$ are (zero-time) inverters, and ${\mathrm{Chan}}_{1}$ and ${\mathrm{Chan}}_{2}$ are (binary) fixed-delay channels with delay d>0. We connect the modules' ports sequentially, as depicted in Fig. 3: The input port *i* of InvChain is also the input port of ${\mathrm{Inv}}_{1}$, whose output port *a* is the input port of ${\mathrm{Chan}}_{1}$; the output *b* of ${\mathrm{Chan}}_{1}$ is fed into ${\mathrm{Inv}}_{2}$, whose output *c* in turn is input to ${\mathrm{Chan}}_{2}$; finally, the output port *o* of ${\mathrm{Chan}}_{2}$ is also the output port of *M*.

Let $E_{\text{in}}$ be the execution of port *i* whose state function is i(t)=0 for t∈(−∞,0) and i(t)=1 for t∈[0,∞). For the module specification of InvChain's corresponding basic module β(InvChain) it then holds that $\Phi_{\beta (\mathrm{InvChain})}(E_{\text{in}})=\{E_{\text{out}}\}$, where $E_{\text{out}}$ is the execution of port *o* with o(t)=0 for t∈(−∞,2d) and o(t)=1 for t∈[2d,∞).

Note that $\Phi_{\beta (\mathrm{InvChain})}$ is extendable: Any feasible execution E on some interval $\left[t^{-},t^{+}\right)$ satisfies o(t)=c(t−d)=¬b(t−d)=¬a(t−2d)=i(t−2d) for all times $t\in [t^{-}+2d,t^{+})$. An infinite feasible execution of InvChain on R whose restriction to $\left[t^{-},t^{+}\right)$ matches E is easily found by (i) extending the input ports execution to $\left[t^{-}-2d,t^{-}\right)$ by using i(t−2d)=o(t) on $\left[t^{-},t^{-}+2d\right)$, (ii) extending the output ports execution to $\left[t^{+},t^{+}+2d\right)$ by using o(t+2d)=i(t) on $\left[t^{+}-2d,t^{+}\right)$, and finally defining o(t)=i(t−2d) arbitrarily for $t\in (-\infty ,t^{-})$ and $t\in [t^{+}+2d,\infty )$.

From this observation, we can infer by Lemma 3.3 that the module specification of InvChain matches that of a fixed-delay channel with delay 2*d*, as in feasible executions of InvChain on R we clearly have o(t)=i(t−2d) at all times t∈R. This demonstrates that compound modules permit to reason about the behavior of complex systems in a hierarchical fashion, based on basic modules that can be understood much easier. The level of detail can be adjusted by the granularity at which one relies on basic modules; in particular, it is feasible to refine the analysis later on by further breaking down basic modules into compound modules.

Example 3.6 As an example of a non-extendable specification, consider the compound module Osc, a simple oscillator that is started at, say, time 0. The module has no input port and one (binary) output port *c*, the clock signal.^14^ It comprises a binary *resettable fixed-delay channel* (RChan) of delay d>0, with input port *c* and output port *o*, whose extendable specification demands that its output fulfills o(t)=0 for all times t∈(−∞,0) and o(t)=c(t−d) for all times t∈[0,∞). Port *o* further is the input port of a (zero-time) inverter (Inv), whose output port is *c*, the clock signal.

It is not hard to see that the only feasible execution of Osc on R maintains signal 0 on port *o* and signal 1 on port *c* until time 0. Then the feedback loop starts to oscillate with frequency 1/(2d), since the channel reproduces its input with fixed delay *d* and the inverter inverts the signal. The specification of Osc is not extendable. This can be seen from the execution on [0,∞) where o(t)=kmod2 for all t∈[kd/3,(k+1)d/3) and $k\in N_{0}$, and c(t)=¬o(t) for all t∈[0,∞). This execution is feasible when restricted to each submodule since c(t)=¬o(t)=o(t−d) for all t∈[d,∞), but the output signal c(t) oscillates at frequency 3/(2d). Hence this execution is feasible, but not a restriction of the unique feasible execution of Osc on R.

**Fig. 4:**
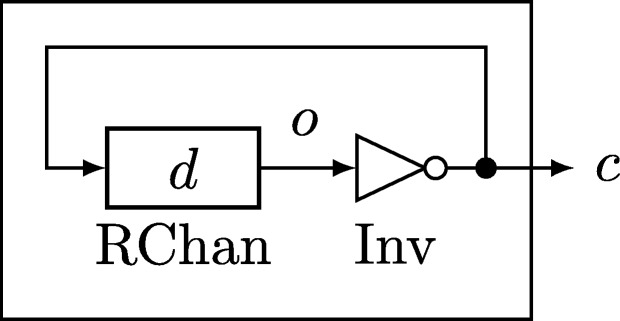
Compound module Osc.

Note carefully that an oscillator according to Fig. 4 involving a channel with delay d=0 would be an example of a well-formed set of modules *M* that is *not* a compound module, since it violates non-emptyness of β(M).

### 3.3 Forgetfulness

Examples 3.5 and 3.6 of InvChain and Osc reveal a crucial insight about the behavior of compound modules. While InvChain behaves like a fixed-delay channel in *all* feasible executions, it cannot be said for Osc that it always behaves like an oscillator of frequency 1/(2d). Even though Osc will run at the fixed frequency of 1/(2d) if it is feasible at all times, an inconsistent initialization or a transient fault can cause it to run forever at an arbitrarily high frequency. In general, when devising compound modules, typically our design goal will be to implement a certain basic module, that is, essentially ensure that the feasible executions of the compound module are also feasible according to the (usually more idealized) specification of the respective basic module. For InvChain, the intended basic module is a fixed-delay channel, where for Osc we had an (externally started) oscillator of frequency 1/(2d) in mind. Since we strive for self-stabilization properties, intuitively InvChain would be a “good” implementation of a fixed-delay channel, whereas Osc is not satisfactory because it does not recover from transient faults.

Before we formalize the concepts of implementation and self-stabilization, let us get a better understanding of the critical difference between InvChain and Osc. In both cases, the output depends on past inputs, so both compound modules have a sort of memory. In any real-world system, this cannot be avoided since physics entails positive delays for any building block we might use. However, InvChain eventually “forgets” about previous inputs, while Osc contains a feedback-loop that, in a feasible execution, determines the future output as a periodic function of the first *d* time units of the execution.

This motivates the following definitions. For $F\in R_{0}^{+}$, we say that a module is *F-forgetful*, iff its specification permits the following construction:

1. Take an arbitrary feasible execution E of *M* on some interval $[t^{-},t^{+}]\subseteq R$. Denote by $E_{\text{in}}$ and $E_{\text{out}}$ its restrictions to the input and output ports of *M*, respectively.
2. Restrict $E_{\text{out}}$ to $E_{\text{out}}^{\prime }$ on $\left[t^{-}+F,t^{+}\right)$. Then for each execution $E_{\text{in}}^{\prime }$ on R whose restriction to $\left[t^{-},t^{+}\right]$ equals $E_{\text{in}}$, there is a feasible execution of *M* on R whose respective restrictions to the input and output ports (and in time) are $E_{\text{in}}^{\prime }$ and $E_{\text{out}}^{\prime }$.

We say a module is *forgetful* iff it is *F*-forgetful for some $F\in R_{0}^{+}$. Essentially, *F*-forgetfulness requires that feasibility of the output at time *t* merely depends on the inputs during [t−F,t]. That is, the effects of earlier inputs, as well as all traces of a possible transient fault, wear out from the internal state within time *F*. Note that 0-forgetful modules are exactly those with extendable specifications, i.e., forgetfulness is a generalization of extendability.

Let the *circuit graph* of a compound module *M* be the directed graph whose nodes are the submodules $S_{M}$, and for each port *p* that is an output port of $S\in S_{M}$ and an input port of $S^{\prime }\in S_{M}$ it contains a (directed) edge from *S* to $S^{\prime }$. We recursively define that *M* is *feedback-free* iff (i) all its basic submodules are forgetful, (ii) all its compound submodules are feedback-free, and (iii) its circuit graph is acyclic. Finally, we define the *idealized basic module* ι(M) corresponding to compound module *M* (with the same ports as *M*) by execution E of ι(M) being feasible iff it is the restriction of a feasible execution of *M* on R.

According to these definitions, InvChain is clearly 2*d*-forgetful and feedback-free. Since we observed that $\Phi_{\beta (\mathrm{InvChain})}$ is extendable, it is in fact 0-forgetful and its corresponding basic and idealized basic modules are identical. In contrast, Osc satisfies neither of these properties. Note, however, that replacing a basic module in InvChain by a compound module might result in a module that does not have these properties either.

We are now in the position to formulate a theorem that states that any feedback-free module will eventually behave like its idealized basic module in a feasible execution.

Theorem 3.7 *Suppose M is a feedback-free compound module. Let* P *be the set of paths in its circuit graph and submodule* $S\in S_{M}$ *be* $F_{S}$*-forgetful. Then M is F-forgetful with*

$$F:=\max_{(S_{1},\ldots ,S_{k})\in P}\left\{\sum_{i=1}^{k}F_{S_{i}}\right\}.$$

Proof Consider an arbitrary execution E on $\left[t^{-},t^{+}\right)$ on the ports of *M* and its submodules whose restriction to the ports of *M* (and thus also its restrictions to each submodule of *M*) is feasible, its restrictions $E_{\text{in}}$ and $E_{\text{out}}$ to the input and output ports of *M*, the restriction $E_{\text{out}}^{\prime }$ of $E_{\text{out}}$ to $\left[t^{-}+F,t^{+}\right)$, and an arbitrary execution $E_{\text{in}}^{\prime }$ on R satisfying that its restriction to $\left[t^{-},t^{+}\right)$ equals $E_{I}$. Denote for each $S\in S_{M}$

$$F_{S,M}:=\max_{(S_{1},S_{2},\ldots ,S_{k}=S)\in P}\left\{\sum_{i=1}^{k}F_{S_{i}}\right\}.$$

Note that $\max_{S\in S_{M}}\{F_{S,M}\}=F$.

By induction on the submodules of *M*, we will construct a feasible execution $E_{i_{\max}}$ on the ports of *M* and its submodules on R that is feasible for *M* when restricted to the ports of *M* and whose respective restrictions equal $E_{\text{in}}^{\prime }$ and $E_{\text{out}}^{\prime }$. In each step of the induction, we will add the output ports of some submodule of *M* to the execution. The induction halts once we run out of submodules of *M* after $i_{\max}⩽|S_{M}|$ steps. The induction hypothesis states for the execution $E_{i}$ on the union of the input ports of *M* and the ports of a subset of its submodules that

• its respective restrictions to submodules are feasible,
• its restriction to the input ports of *M* equals $E_{\text{in}}^{\prime }$,
• its restriction to output ports of *M* on $\left[t^{-}+F,t^{+}\right)$ matches the restriction of $E_{\text{out}}^{\prime }$ to such ports, and
• for each submodule $S\in S_{M}$ whose execution $E_{S}$ is already fully specified by $E_{i}$, we have that $E_{S}$ restricted to $\left[t^{-}+F_{S,M},t^{+})⊇[t^{-}+F,t^{+}\right)$ equals the corresponding restriction of E.

Note that these properties together prove the theorem, since they show the claims we made on the properties of $E_{i_{\max}}$, and E and $E_{\text{in}}$ are chosen arbitrarily (respecting the constraints imposed by the definition of forgetfulness). We set $E_{0}:=E_{\text{in}}^{\prime }$ and anchor the induction at $E_{0}$, trivially satisfying the induction hypothesis for i=0 and guaranteeing that all $E_{i}$ restricted to the input ports of *M* equal $E_{\text{in}}^{\prime }$.^15^ Recall that the input ports of *M* cannot be output ports of its submodules. Any other ports of *M* and its submodules are output ports of a submodule, hence the final execution will indeed contain all ports and thus cover all submodules.

To perform the induction step, suppose we have already constructed $E_{i-1}$ for some $i\in [1..i_{\max}]$. By definition of $i_{\max}$, there is an uncovered submodule left, i.e., $E_{i-1}$ does not specify the execution on all ports of all submodules. Because *M* is feedback-free, its circuit graph is acyclic. Consider the subgraph of the circuit graph induced by the remaining uncovered submodules. As it is acyclic as well, there must be a module *S* without an incoming edge, implying that $E_{i-1}$ specifies executions of all its input ports. By the induction hypothesis, we have that these executions restricted to $\left[t^{-}+F_{S,M}-F_{S},t^{+}\right)$ equal the respective restrictions of E: Any such port is either an input port of *M* and therefore its executions in $E_{i-1}$ and E are identical on $\left[t^{-},t^{+}\right)$, or it is an output port of some module $S^{\prime }$ satisfying that there is some path $(S_{1},\ldots ,S_{k-1}=S^{\prime },S_{k}=S)\in P$ and therefore the executions of the port in $E_{i-1}$ and E are identical on $\left[t^{-}+F_{S^{\prime },M},t^{+})⊇[t^{-}+F_{S,M}-F_{S},t^{+}\right)$. Since the output ports of *S* cannot be output ports of other modules and the input ports of *M* cannot be output ports of submodules, $E_{i-1}$ does not specify executions for any of the output ports of *S*. We apply that *S* is $F_{S}$-forgetful to the restriction of E to the ports of *S* on the interval $\left[t^{-}+F_{S,M}-F_{S},t^{+}\right)$ and the input execution given by the restriction of $E_{i-1}$ to the input ports of *S*, which is possible since we observed that the restrictions of these executions to the input ports of *S* on $\left[t^{-}+F_{S,M}-F_{S},t^{+}\right)$ are identical. We obtain a feasible execution of *S* on R whose restriction to the input ports matches the restriction of $E_{i-1}$ to these ports and whose restriction to the output ports and $\left[t^{-}+F_{S,M},t^{+}\right)$ matches the respective restriction of E. We define $E_{i}$ by adding these executions of the output ports of *S* to $E_{i-1}$. Overall, the first, second, and fourth claim of the induction hypothesis thus hold by construction. Taking into account that $\left[t^{-}+F_{S,M},t^{+})⊇[t^{-}+F,t^{+}\right)$ and $E_{\text{out}}^{\prime }$ is a restriction of E, the same holds for the third claim. Hence the induction step succeeds, finishing the proof.  □

As a consequence, any feedback-free compound module will eventually “forget” about transient faults and behave according to *some* fault-free history. Note that in general that does not mean that if the module's execution is feasible on two separate intervals there is any guaranteed relation between the behavior on these intervals, as arbitrary transient faults result in arbitrary modifications of the module's state. However, we can assume that such a module, once it becomes non-faulty and thus follows its specification, eventually behaves correctly in the strong sense given by the specification of its corresponding idealized basic module.

The bad news is that we have to introduce feedback-loops into the system at some point: After all, any clock source is some kind of oscillator. As demonstrated by Osc, we cannot expect a strong generic result like Theorem 3.7 for compound modules that are not feedback-free. Also, clearly such a structure cannot be forgetful. Hence, let us turn to a less restrictive concept of recovery from transient faults.

### 3.4 Self-stabilization

Self-stabilization is the property of a system to recover from arbitrary transient faults in finite time, provided that all transient faults cease. Since arbitrary transient faults may result in arbitrary states, this requirement can be rephrased as the need to reach a valid state within finite time from any initial state. For basic modules, our framework encapsulates this concept by the notion of feasibility; executions completely hide the internal state of a basic module, and we assume (or hope) that the utilized implementation of the module will recover from faults that are considered transient, e.g. particle hits, crosstalk, magnetic fields, or power outages. For compound modules, one possible meaning of “valid state” in our context is given by the specification of the corresponding idealized basic module: If, viewed from the outside, the execution is indistinguishable from one that could occur if the module was entirely fault-free, we can safely assume that its state is valid; any fault that is masked is irrelevant to the higher layers of the system anyway, as it solely relies on the guarantees of the utilized model specification on the module's ports' executions.

A more general concretization of the same underlying idea is the notion of self-stabilization we present next. It introduces two relaxations of the constraints on the behavior of a module. Firstly, we do not expect that the recovering module is perceived as functional by an outsider immediately after its execution becomes feasible. Rather, we allow for some *stabilization time* during which the module can “clean up” its internal state. Secondly, we do not require that the module fulfills its corresponding idealized basic module specification. Instead, we choose another, weaker specification that is sufficient for our purposes. One specification being stronger than another is captured by the following notion. We define that module *M implements* module $M^{\prime }$ iff both modules have the same sets of input and output ports and $\Phi_{M}(E_{\text{in}})\subseteq \Phi_{M^{\prime }}(E_{\text{in}})$ for all $E_{\text{in}}$. Thus, $\Phi_{M}$ is stronger than $\Phi_{M^{\prime }}$ in the sense that it imposes at least as many constraints on the output executions as $\Phi_{M^{\prime }}$. For $T\in R_{0}^{+}$, module *M* now is a *T-stabilizing implementation of module* $M^{\prime }$, iff the restriction of each feasible execution of *M* on $[t^{-},t^{+})\subseteq R$ to $\left[t^{-}+T,t^{+}\right)$ is a feasible execution of $M^{\prime }$. Clearly, for T>0, this allows for $\Phi_{M}(E_{\text{in}})⊈\Phi_{M^{\prime }}(E_{\text{in}})$, i.e., a *T*-stabilizing implementation of $M^{\prime }$ needs not be an implementation of $M^{\prime }$. For brevity, we may say that “*M* is *T*-stabilizing” to express that *M* is a *T*-stabilizing implementation of its corresponding idealized basic module. We simply say *M* is a *self-stabilizing* (implementation of $M^{\prime }$) iff it is a *T*-stabilizing (implementation of $M^{\prime }$) for some $T\in R_{0}^{+}$.

From our previous definitions and results, we immediately can derive the following statements.

Lemma 3.8

1. *Every F-forgetful module is F-stabilizing.*
2. *Every feedback-free compound module is self-stabilizing.*
3. *If M is a self-stabilizing implementation of* $M^{\prime }$ *and* $\Phi_{M}$ *is extendable, then M is an implementation of* $M^{\prime }$*.*
4. *Every feedback-free compound module whose specification is extendable implements its corresponding idealized basic module.*

Proof The first statement follows directly from the definition of *F*-forgetfulness. The second statement follows from Theorem 3.7 and the first statement. Regarding the third statement, by extendability every feasible execution of *M* on some interval I⊆R is the restriction of a feasible execution E of *M* on R. By the definition of self-stabilizing implementations, there is some $T\in R_{0}^{+}$ such that E restricted to (−∞+T,∞)=R is feasible for $M^{\prime }$. By properness, restricting E to *I* yields a feasible execution of $M^{\prime }$. Since this restriction equals the original feasible execution of *M*, it follows that every feasible execution of *M* is a feasible execution of $M^{\prime }$. The last statement follows from the second and third.  □

Recall that InvChain from Example 3.5 behaves like a fixed-delay channel with delay 2*d*. As InvChain is feedback-free and its specification is extendable, we could also infer this statement directly from Lemma 3.8. In contrast, Osc is not self-stabilizing at all, i.e., it is not self-stabilizing for any $T\in R_{0}^{+}$, which follows from the same arguments that we used to show that its specification is not extendable. In general, determining whether a module *M* is a self-stabilizing implementation of some other module $M^{\prime }$ (or even bounding the stabilization time) requires a detailed stabilization analysis.

One may as well generalize the above definition to also account for probabilistic stabilization by defining an appropriate probability space on the set of executions of *M*. For the sake of brevity and better readability, we only informally state the probability space in this work by introducing basic modules that act in a probabilistic manner, namely the Randomized Watchdog Timers. Probabilistic bounds on the stabilization time of compound implementations then are naturally derived from the respective distributions of their submodules. The interested reader is referred to [13], where we give a precise definition of the probability space over which our probabilistic stabilization guarantees are expressed.

### 3.5 Persistent faults

We next generalize the definition of feasibility for compound modules to potentially faulty submodules. For a compound module *M* and f⩾0, we define the *corresponding f-faulty basic module* $\beta_{f}(M)$ as follows: $\beta_{f}(M)$'s input and output ports are the input and output ports of *M*. Let E be any execution of *M* on I⊆R and denote by $E_{\text{in}}$ and $E_{\text{out}}$ its restrictions to the input and output ports of *M*, respectively. Then $E_{\text{out}}\in \Phi_{\beta_{f}(M)}(E_{\text{in}})$, iff there exists an execution $E_{M,S_{M}}$ of all ports of modules in $\{M\}\cup S_{M}$ and a subset F of $S_{M}$ of size at most *f*, such that, for each module *S* in $S_{M}\setminus F$, the restriction of execution $E_{M,S_{M}}$ to the ports of *S* is feasible, and $E_{M,S_{M}}$ restricted to *M*'s input and output ports is equal to E. As in the fault-free case, we require that $\Phi_{\beta_{f}(M)}$ fulfills non-emptiness. For compound module *M* and f⩾0, we say an execution E of *M* is *f-faulty feasible* iff it is feasible for the corresponding *f*-faulty basic module $\beta_{f}(M)$. Modules whose behavior is unrestricted in execution E (i.e., that belong to the set F) are called *faulty (in execution* E*)*.

Clearly, a compound module *M* cannot mask faults of submodules that have an output port that is also output port of *M*. Consequently, tolerance of ongoing faults necessitates to weaken the constraints on *M*'s output. Hence, for f⩾0, compound module *M*, and some other module $M^{\prime }$ with the same set of input and output ports, we say that *M* is an *f-tolerant implementation of* $M^{\prime }$ iff every *f*-faulty feasible execution of *M* is a feasible execution of $M^{\prime }$, i.e., the corresponding *f*-faulty basic module of *M* implements $M^{\prime }$. Analogously, *M* is an *f-tolerant T-stabilizing implementation of* $M^{\prime }$ iff its corresponding *f*-faulty basic module is a *T*-stabilizing implementation of $M^{\prime }$.

Note that this entails that the union of output ports of any set consisting of *f* submodules of *M* can be arbitrary. The module specification of $M^{\prime }$ thus must not put concurrent restrictions on all its output ports to allow for fault-tolerance. Hence, one demands constraints on the outputs of non-faulty submodules of *M* (i.e., those whose executions are feasible) only.

## 4 Formal specification of clock synchronization protocols

**Fig. 5:**
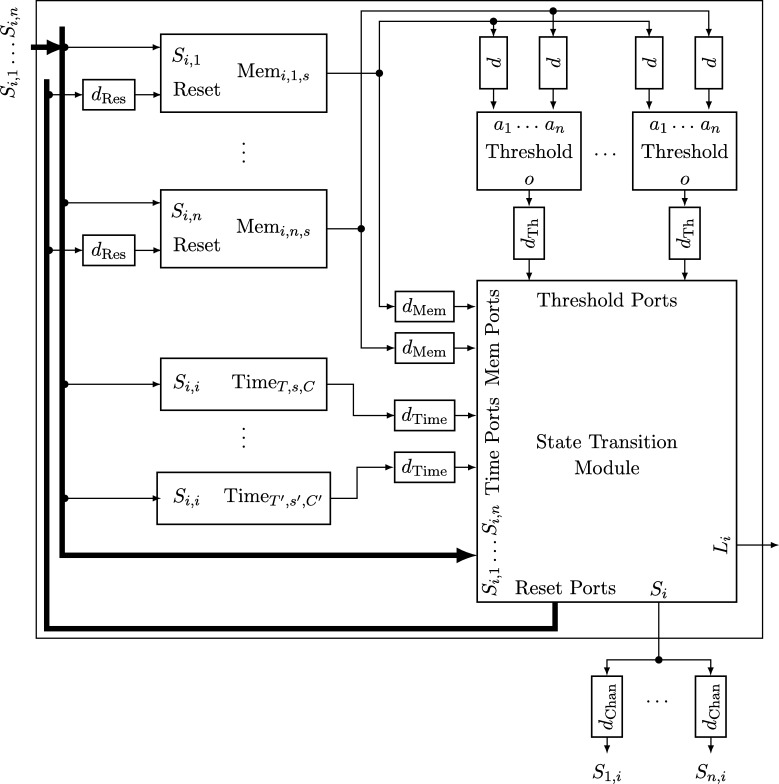
Submodules of node *i* and their interconnection.

In the formal framework introduced above, a *node* is simply a compound module that will operate according to some specification whenever it is non-faulty. We now specify the submodules of a node introduced informally in Section 2.2 and reveal how they are connected. The reader might want to take a look at Fig. 5 to get an idea of the general layout of a node at this point. Each node i∈[1..n] has *n* input ports $S_{i,j}$, where j∈[1..n], and n+1 output ports, namely $L_{i}$ and $S_{j,i}$ for all j∈[1..n]. We present all submodules as basic modules whose specifications are sufficiently strong to implement the model used in [13]. As a result, not all specifications can be satisfied by (physical) implementations of the modules for all input executions; we discuss these limitations and their implications in Section 6. All of the following specifications are extendable and therefore, by Lemma 3.3, are uniquely characterized by describing them on executions on R only. It is trivial to verify non-emptyness for the given specifications, hence omit respective discussions.

• **Communication channels.** For each j∈[1..n], there is a communication channel of delay $d_{\mathrm{Chan}}\in R^{+}$ from node *i*'s internal port $S_{i}$ to its output port $S_{j,i}$. Formally, this communication channel is a basic module with input port $S_{i}$ and output port $S_{j,i}$. The module specification $\Phi_{\mathrm{Chan}}$ of the communication channel is as follows. Let $E_{\text{in}}$ be an execution of input port $S_{i}$ and $E_{\text{out}}$ an execution of output port $S_{j,i}$. Then $E_{\text{out}}\in \Phi_{\mathrm{Chan}}(E_{\text{in}})$ iff there exists a continuous, strictly increasing (and thus invertible) *delay function* τ:R→R such that for all t∈R both (i) $S_{j,i}(t)=S_{i}(\tau^{-1}(t))$ and (ii) $0⩽t-\tau^{-1}(t)<d_{\mathrm{Chan}}$ hold.
  It is important to note that the assumptions on the communication channels are strictly weaker than those on fixed-delay channels, as the delay of a communication channel may vary arbitrarily (within bounds) during an execution.
• **Memory flags.** For each state s∈S and each node j∈[1..n], there is a memory flag ${\mathrm{Mem}}_{i,j,s}$ at node *i*. It has two input ports, namely $S_{i,j}$ and a binary reset port, as well as a binary output port ${\mathrm{Mem}}_{i,j,s}$ whose name is for simplicity identical to the memory flag's name. Given an execution $E_{\text{in}}$ of the flag's input ports, an execution $E_{\text{out}}$ of the output port is in $\Phi_{\mathrm{Mem}}(E_{\text{in}})$ iff properties (Reset) and (Set) hold:
  – *(Reset)* For all times t∈R, ${\mathrm{Mem}}_{i,j,s}(t)=0$ iff the reset port has been in state 1 at some time between $\sup\{t^{\prime }\in (-\infty ,t]|S_{i,j}(t^{\prime })=s\}$ and *t*.
  – *(Set)* For all times t∈R, ${\mathrm{Mem}}_{i,j,s}(t)=1$ iff the reset port continuously has been in state 0 between $\sup\{t^{\prime }\in (-\infty ,t]|S_{i,j}(t^{\prime })=s\}$ and *t*.
  We say that node *i memorizes node j in state s* at time *t* iff ${\mathrm{Mem}}_{i,j,s}(t)=1$.
• **Threshold gates.** Node *i* may comprise an arbitrary number of threshold gates with arbitrary thresholds. The module specification $\Phi_{\mathrm{Thr}}$ of a threshold gate with binary input ports $a_{1},\ldots ,a_{m}$, binary output port *o*, and threshold k∈[1..m] is defined as follows. Let $E_{\text{in}}$ be an execution of the input ports and $E_{\text{out}}$ an execution of output port *o*. Then $E_{\text{out}}\in \Phi_{\mathrm{Thr}}(E_{I})$ iff for all t∈R, o(t)=1 if at least *k* input ports are in state 1 at time *t*, and o(t)=0 otherwise.
• **Watchdog timers.** For each state s∈S, there can be watchdog timers (T,s,C) at node *i*, where $T\in R^{+}$ is the *duration* of the timer and *C* is a clock. The watchdog timer has input port $S_{i,i}$ and a binary output port ${\mathrm{Time}}_{T,s,C}$. The timer's module specification $\Phi_{\mathrm{Time}}$ is defined as follows. Let $E_{\text{in}}$ be an execution of the timer's input port $S_{i,i}$ and $E_{\text{out}}$ an execution of its output port ${\mathrm{Time}}_{T,s,C}$. Then $E_{\text{out}}\in \Phi_{\mathrm{Time}}(E_{\text{in}})$, iff the following holds:
  – *(Clock)* Clock *C* is correct at all times, i.e., $t^{\prime }-t⩽C(t^{\prime })-C(t)⩽\vartheta (t^{\prime }-t)$ for all $t,t^{\prime }\in R$, $t<t^{\prime }$.
  – *(Reset)* There exists a (binary) signal $\sigma_{T,s,C}\in [{\mathrm{Time}}_{T,s,C}]$ (the equivalence class of the output port's signal) such that, for each time $t_{s}\in R$ when $S_{i,i}$ switches to state *s*, there is a time $t\in [t_{s},t_{s}+d_{\mathrm{Time}}]$ such that (T,s,C) is *reset*, i.e., event $(0,t)\in \sigma_{T,s,C}$. This is a one-to-one correspondence, i.e., for each such $t_{s}$ this time *t* is unique and (T,s,C) is not reset at any other times.
  – *(Expire)* Denote by R⊂R the set of times when (T,s,C) is reset. For each time $t_{R}\in R$, denote by $t_{E}(t_{R})$ the unique time satisfying that $C(t_{E}(t_{R}))-C(t_{R})=T$. Then, for each t∈R, ${\mathrm{Time}}_{T,s,C}(t)=0$ iff $t\in {⋃}_{t_{R}\in R}[t_{R},t_{E}(t_{R}))$.
  Iff $(t_{E}(t_{R}),1)\in {\mathrm{Time}}_{T,s,C}$, i.e., (T,s,C) is not reset during $\left(t_{R},t_{E}(t_{R})\right]$ again and hence ${\mathrm{Time}}_{T,s,C}$ switches to 1 at time $t_{E}(t_{R})$, we say that (T,s,C) *expires* at time $t_{E}(t_{R})$. (T,s,C) *is expired* at time t∈R iff ${\mathrm{Time}}_{T,s,C}(t)=1$. For notational convenience, we will omit the clock *C* and simply write (T,s) for both the timeout and its signal.
• **Randomized watchdog timers.** A randomized watchdog timer (D,s,C) is a module with input port $S_{i,i}$ and output port ${\mathrm{Time}}_{D,s,C}$, where D is a bounded random distribution on $(0,D]\subset R^{+}$, *s* is a state, and *C* a clock. The module specification of (D,s,C) is analogous to the module specification of a watchdog timer, except that property *(Expire)* is replaced by:
  – *(Expire')* Denote by R⊂R the set of times when (T,s,C) is reset. For each time $t_{R}\in R$, denote by $t_{E}(t_{R})$ the unique time satisfying that $C(t_{E})-C(t_{R})=T(t_{R})$, where $T(t_{R})$ is drawn (independently) from D. Then, for each t∈R, ${\mathrm{Time}}_{T,s,C}(t)=0$ iff $t\in {⋃}_{t_{R}\in R}[t_{R},t_{E}(t_{R}))$.
  We apply the same notational conventions as for watchdog timers.
• **State transition modules.** Node *i*'s state transition module has input ports $S_{i,j}$ for each node j∈[1..n] as well as one binary input port for each of the memory flags, (randomized) watchdog timers and threshold gates it uses. Furthermore it has an output port $S_{i}$ as well as one binary Reset output port for each of the memory flags it uses.
  A node's state transition module executes a state machine specified by (i) a finite set S of states, (ii) a function *tr*, called the *transition function*, from $T\subseteq S^{2}$ to the set of Boolean predicates on the alphabet consisting of expressions of the form “p=s” (used for expressing guards), where *p* is from the state transition module's input ports and *s* is from the set of possible states of signal *p*, and (iii) a function *re*, called the *reset function*, from T to the power set of the node's memory flags.
  Intuitively, the transition function specifies the conditions (guards) under which a node switches states, and the reset function determines which memory flags to reset upon the state change. Formally, let *P* be a predicate on the input ports of node *i*'s state transition module. We define *P holds at time t* by structural induction: If *P* is equal to p=s, where *p* is an input port of node *i*'s state transition module and *s* is one of the states signal *p* can obtain, then *P holds at time t* iff p(t)=s. Otherwise, if *P* is of the form $\neg P_{1}$, $P_{1}\wedge P_{2}$, or $P_{1}\vee P_{2}$, we define *P holds at time t* in the straightforward manner.
  For a given transition delay $d_{\mathrm{Trans}}>0$, the module specification $\Phi_{\mathrm{STM}}$ of node *i*'s state transition module is defined as follows. Let $E_{\text{in}}$ be an execution of the state transition module's input ports and $E_{\text{out}}$ an execution of its output ports. Then $E_{\text{out}}\in \Phi_{\mathrm{STM}}(E_{\text{in}})$ iff there is some ε>0 and a signal locked such that the following requirements are met. (The intuition is that locked(t)=0 means that the node is ready to perform the next state transition once a guard becomes true, whereas in case of locked(t)=1 the node is currently executing a previously “locked” transition.)
  – *(Safety)* The node (i.e., $S_{i}$) does not switch states at any time *t* with locked(t)=0. In every maximal interval $[t_{l},t_{u})\subseteq R$ satisfying that locked≡1 on $\left[t_{l},t_{u}\right)$, it switches states exactly once.
  – *(Delay)* For each interval $\left[t_{l},t_{u}\right)$ as above, $t_{u}-t_{l}⩽d_{\mathrm{Trans}}-\varepsilon$.
  – *(Guard)* For each interval $\left[t_{l},t_{u}\right)$ as above, $(S_{i}(t_{l}),S_{i}(t_{u}))\in T$ and $tr(S_{i}(t),S_{i}(t_{u}))$ is satisfied at some time $t\in [t_{l}-\varepsilon ,t_{l}]$.
  – *(Responsiveness)* If locked(t)=0 and there is a state s∈S such that $(S_{i}(t),s)\in T$ and $tr(S_{i}(t),s)$ holds at time *t*, then locked(t+ε)=1.
  – *(Flags)* For an arbitrary interval $\left[t_{l},t_{u}\right)$ as above, suppose that the node switches from state $S_{i}(t_{l})$ to state $S_{i}(t_{u})$ at time $t_{s}\in [t_{l},t_{u})$. Then for each memory flag specified by $re(S_{i}(t_{l}),S_{i}(t_{u}))$, the corresponding reset output port of the state transition module is in state 1 at some time in $\left(t_{l},t_{s}\right]$ (and therefore the flag is reset). Outside these time intervals, reset ports are in state 0.
  A node may run multiple, say k∈N, state machines in parallel (i.e., contain several state machines as submodules). In this case, its state signal is the joint signal $S_{i}=(S_{i}^{1},\ldots ,S_{i}^{k})$, where $S_{i}^{l}$, l∈[1..k], denotes the $l^{th}$ state machine of the node. Throughout this article, the different state machines of each node *i* have disjoint state spaces. For simplicity, we hence may say “node *i* is in state *s* at time *t*” instead of “state machine *l* of node *i* is in state *s* at time *t*” when referring to $S_{i}^{l}(t)=s$, etc.

To account for the latency of the memory flags, threshold gates and (randomized) watchdog timers, their ports are not directly connected to the state transition module's ports, but via binary communication channels with respective delays. The resulting structure of the compound module node *i* is depicted in Fig. 5. Note that additional communication channels at the threshold gates' and memory flags' input ports allow to model the fact that memory flags are not necessarily reset at the same time, and signals may arrive shifted in time at the threshold gates.

As mentioned earlier, for simplicity we consider the outgoing channels to remote nodes as part of the node. Hence, the output ports of node *i* comprise the output ports $S_{j,i}$, j∈[1..n], of the channels disseminating its state $S_{i}$. In addition, in order to solve the actual problem of clock generation, we include the locally computed discrete clock value $L_{i}$ as an output port.

### 4.1 Protocols and problem formulation

We next formalize the concept of a protocol, like the one presented in Section 5, followed by what it means for a protocol to solve self-stabilizing clock synchronization in spite of *f* faults. Formally, a *protocol (for an n-node system)* is a compound module consisting of *n* modules referred to as nodes. The nodes are to be specified as modules themselves, and in our case will follow the layout we just described. It thus remains to state in Section 5 which (randomized) watchdog timers, memory flags and threshold gates our protocol uses as well as the state transition modules' transition and reset functions.

A *clock synchronization module with* n∈N *nodes, clock imprecision Σ, amortized frequency bounds* $A^{-},A^{+}\in R^{+}$*, slacks* $\tau^{-},\tau^{+}\in R_{0}^{+}$*, maximum frequency* $F^{+}$*, and at most* f∈N *faults* is a module without input ports and with output ports $L_{i}$, i∈[1..n]. Its module specification is extendable. An execution of the module on R is feasible, iff there exists a subset C of [1..n] of size at least n−f satisfying that

• ∀t∈R, i,j∈C: $|L_{i}(t)-L_{j}(t)|⩽\Sigma$,
• ∀*t*, $t^{\prime }\in R$, $t<t^{\prime }$, i∈C: $A^{-}(t^{\prime }-t)-\tau^{-}⩽L_{i}(t^{\prime })-L_{i}(t)⩽A^{+}(t^{\prime }-t)+\tau^{+}$, and
• ∀*t*, $t^{\prime }\in R,t<t^{\prime }$, i∈C: $L_{i}(t^{\prime })-L_{i}(t)⩽\lceil F^{+}(t^{\prime }-t)\rceil$.

We say a protocol *Π* (for an *n*-node system) with no input ports and output ports $L_{i}$, i∈[1..n], *solves self-stabilizing clock synchronization with clock imprecision Σ, amortized frequency bounds* $A^{-},A^{+}$*, slacks* $\tau^{-},\tau^{+}\in R_{0}^{+}$*, maximum frequency* $F^{+}$*, at most f faults, and stabilization time T* (*with probability p*) iff it is an *f*-tolerant, (with probability at least *p*) *T*-stabilizing implementation of the clock synchronization module with the respective parameters.

A (real-world) implementation will output *bounded clocks of size* K∈N only. In this case the output ports do not yield $L_{i}(t)$, but only $L_{i}(t)\mathrm{mod}\;K$. Nevertheless, we introduced the signals $L_{i}$ as abstract functions in this setting, as they allow to state the frequency bounds concisely. Note that there is no physical counterpart of these values in the real-world system; to be strictly accurate, it would be necessary to qualify the above definitions further by “with bounded clocks of size *K*” in order to distinguish this version of the problem from the abstract one with unbounded clocks.

### 4.2 Practical implementability issues

Our formal model incorporates a precise semantics of what it means for a module to implement some other module, namely, inclusion of *all* feasible (sub-)executions. Unfortunately, however, this strong requirement must often be relaxed when it comes to real implementations. This is primarily a consequence of the fact that there is no physical implementation of a circuit that can avoid *metastability*. Since preventing certain inputs to a module requires output guarantees from others, this is a challenging problem to systems that are self-stabilizing *or* tolerate persistent faults; combining these properties complicates this issue further.

More specifically, in order to faithfully implement their specifications, basic modules must be able to (i) deal with *all* possible inputs and (ii) recover reliably from transient faults. Unfortunately, (i) is often impossible to achieve with real circuits. For example, simultaneous input changes may drive any implementation of a Muller C-gate into a metastable state, which implies that its output ports do not even carry signals according to our definition, and are hence not feasible. Of course, metastability can also be caused by physical faults affecting the module; such faults can obviously not be analyzed within our model either. This possibility obviously invalidates any guarantees that compound implementations containing this instance may provide, unless they can mask the error due to fault-tolerance. Moreover, real circuits cannot guarantee (ii) under all circumstances either, as it is impossible to always prohibit the propagation of metastable inputs to the outputs and the system may contain feedback-loops.

In principle, it would be possible to extend the presented model to cover also generation and propagation of metastability explicitly, by replacing the finite alphabet S and discrete events with a continuous range of signal values (the voltages) [43]. Since this would dramatically increase the complexity of any analysis, we choose a different approach that also allows us to handle other implementation intricacies in a pragmatic way.

In fact, even in the absence of metastability, it is not necessarily simple and even possible for real implementations to guarantee (ii) under all circumstances. Apart from the fact that transient faults may lead to permanent errors by damaging physical components,^16^ our model does not prohibit that temporarily infeasible inputs result in permanent infeasibility, i.e., even when inputs become benign again at a later state of the execution of the module in question, there is no suffix of the execution that is feasible. The oscillator implementation given in Example 3.6 demonstrates this issue, and further modules exhibiting persistently faulty behavior after temporary violations of input constraints are easily conceived.

As we aim for self-stabilization, it is clear that we cannot allow implementations that suffer from such drawbacks: Neither transient faults nor their consequences, i.e., temporarily arbitrary executions, may result in permanent faults. Clearly, both recovery from transient failures and resilience of a basic module to erroneous inputs, and hence the whole definition of what actually constitutes a transient fault in our model, is implicitly defined by the physical realization of an implementation.

These observations have important consequences. On the one hand, careful design of the basic modules is of paramount importance. For instance, in a final product, a watchdog timer must not have its duration stored in a memory register that can be corrupted by a temporary charge injection (e.g. due to a particle hit), a ring oscillator should not be able to run unchecked at e.g. twice its frequency indefinitely (e.g. triggered by a voltage pulse), and one has to make sure that stateful components like memory flags or state transition modules eventually “forget” about potentially erroneous inputs in the past, and eventually behave according to their specification again. As discussed above, however, this cannot usually be perfect: There will always be (rare) scenarios, where an implemented circuit will not work like an ideal one, i.e., violate its specification. We incorporate this in our model, in a pragmatic well-known from critical system design, by means of the notion of imperfect *implementation coverage*. For a given module implementation, the coverage implicitly or explicitly determines the fraction of all possibly executions in which the implementation works as specified. Since exceptional scenarios like metastability are usually extremely rare, we do not bother with defining the notion of coverage formally here: The coverage should be very close to 100% anyway. In Section 6, we will argue that each of our basic modules will work as specified, except for very rare situations that may trigger metastability due to a violation of input timing constraints.

Thanks to this approach, algorithms and proofs can rely on sufficiently simple specifications of basic modules, which usually also admit robust and efficient implementations in practice. Any unhandled scenarios are relegated to imperfect implementation coverage. This feature is essential for devising proofs of reasonable complexity that show self-stabilization of all compound modules, implying that the system indeed will recover once transient faults of (basic) modules cease. Due to the hierarchical composition of modules, compound modules fully derive their behavior from their submodules and can therefore be analyzed based on the properties of their submodules, while we may switch at will between viewing a module as given (i.e., basic), analyzing it in more detail as a compound implementation, or (for low-level modules) analyzing it in an even more detailed model. This way, our approach also inherently supports tight interaction between algorithmic design and design of the basic building blocks used in the algorithms.

## 5 The FATAL^+^ clock synchronization protocol

In this section, we recast the self-stabilizing clock synchronization algorithm introduced in [13] in the modeling framework of the previous section and summarize its most important properties. Since the main focus of our paper is on the implementation of our algorithm in this model, there is no need to provide a detailed description of the stabilization mechanism, let alone formal proofs of the stated claims; the analysis of the correctness and performance of the algorithm in [13] is based on a simpler abstract system model, assuming a globally valid end-to-end delay bound *d* covering any (local and remote) communication and processing action, which is fully compatible with our modeling framework. More specifically, all that is needed in order to reuse the results of the analysis in [13] is to compute the maximum end-to-end delay occurring in the implementation of our algorithm in the modeling framework introduced in Section 2.2.

Recall from Section 2.2 that our top-level clock synchronization module is implemented as a compound module consisting of *n* nodes and their connecting top-level channels (with maximum delay $d_{\mathrm{Chan}}$). Every node, in turn, is a compound module made up of a state transition module, watchdog timers, memory flags, and threshold modules interconnected by channels (modeling various delays) as shown in Fig. 5. Finally, a state transition module represents several communicating concurrent asynchronous state machines (with maximum transition time $d_{\mathrm{Trans}}$). It ensures that state transitions of every constituent state machine occur in an orderly fashion, i.e., that every transition happens exactly once and, if need be, memory flags are consistently reset. The state of each state machine is encoded in a few bits and conveyed via the top-level channels to all other modules in the system that need to receive it on some input port. Given this simple internal structure, computing the resulting end-to-end delay bound *d* (or, for the quick cycle, $d_{\min}^{+}$ and $d_{\max}^{+}$, see below) from the constituent delay bounds is straightforward, see Section 6 for details.

### 5.1 State machine representation

Obviously, the entire logic of our algorithm is encoded in the state machines of a node. In [13], we use a graphical representation that also reveals the layered structure imposed by their communication. We already employed this description in Fig. 1. With the definitions from the previous section at hand, we can now give our graphical representation a precise formal meaning that will allow us to translate the results from [13] to our modeling framework.

Our graphical representation defines the set of possible states S of a state machine (in Fig. 1 *ready*, *propose*, and *increase*) and, by means of the arrows between the states, the set of possible state transitions $T\subseteq S^{2}$ (here *ready* to *propose*, *propose* to *increase*, and *increase* to *ready*). If, for a state transition from *s* to $s^{\prime }$, $re(s,s^{\prime })\neq \emptyset$, i.e., there are memory flags that need to be reset, $re(s,s^{\prime })$ is given in a rectangular box on the arrow. Since for each node *i* and state *s* we will always reset all memory flags ${\mathrm{Mem}}_{i,j,s}$ for j∈{1,…,n} together, we simply write $s_{1},\ldots ,s_{k}$ in such a box to represent the fact that all flags ${\mathrm{Mem}}_{i,j,s}$, j∈{1,…,n}, $s\in \{s_{1},\ldots ,s_{k}\}$, are to be reset. Note that some of these states may be from a different state machine, i.e., the states $s_{1},\ldots ,s_{k}$ need not all be from S.

Completing the description, for each $(s,s^{\prime })\in T$, $tr(s,s^{\prime })$ is given by the label next to the respective arrow. Again, we make use of a condensed notation. Assume that the state machine in question is part of node *i*. We will employ threshold conditions like “⩾f+1 $s_{1}$”, whereby we refer to at least f+1 of *i*'s memory flags ${\mathrm{Mem}}_{i,j,s_{1}}$ being in state 1, or “⩾n−f $s_{1}$ or $s_{2}$”, which is true if $\sum_{j\in N}\max\{{\mathrm{Mem}}_{i,j,s_{1}},{\mathrm{Mem}}_{i,j,s_{2}}\}⩾n-f$, i.e., for at least n−f nodes *j* flag ${\mathrm{Mem}}_{i,j,s_{1}}$ or flag ${\mathrm{Mem}}_{i,j,s_{2}}$ is in state 1. An example for such a rule is the transition from *propose* to *increase* in Fig. 1. Such conditions will be translated to a binary signal by feeding the memory flags' signals (or, in the latter case, the output of *n* OR-gates with inputs ${\mathrm{Mem}}_{i,j,s_{1}}$ and ${\mathrm{Mem}}_{i,j,s_{2}}$) into a threshold gate (of threshold f+1 or n−f, respectively). Further abbreviations we use for timeouts. Recall that for a timeout (T,s,C), we omit the clock *C* from the notation, i.e., write (T,s) instead of (T,s,C). Timeout (T,s) switches to 1 after *T* local time units (i.e., between T/ϑ and $T+d_{\mathrm{Trans}}$ reference time) has passed since the last switch to state *s* was triggered. In case it is part of a transition rule, we write (T,s) for the condition ${\mathrm{Time}}_{T,s,C}=1$, and if the transition goes from the state *s* to which the timeout corresponds to some state $s^{\prime }$, we simply write *T*. For instance, the condition “3ϑd local time has passed” in Fig. 1 is concisely stated as “3ϑd”.

Finally, as for memory flag resets, transition rules may also refer to a state *s* of another state machine. In the special case that a predicate solely depends on the current state of another of the node's state machines, we write “in *s*” or “not in *s*” to indicate the predicates p=s and ¬(p=s), respectively, where *p* is the input port connected to the channel communicating the other state machine's state to the state transition module. Finally, the above rules can be composed by logical AND or OR, which we display by connecting expressions with **and** or **or**, respectively. In Fig. 1, such a composition occurs in tr(ready,propose).

### 5.2 Overview of the algorithm

Each node is a collection of several state machines that are organized in a layered structure. On each layer, the state machines of the (at least n−f) non-faulty nodes cooperate in order to establish certain synchronization properties of their output signals. The higher is a state machine in the hierarchy, the stronger are these guarantees; the lower it is, the weaker are the synchronization properties its input signals need to satisfy for stabilization. The lowest-layer state machine utilizes randomization to recover from any configuration (provided its basic modules are correct (again), i.e., guarantee feasible executions). Each other layer utilizes auxiliary information from the layer below to stabilize. Finally, the top level state machine outputs the logical clocks $L_{i}$.

More specifically, we have the following state machines.

**Fig. 6:**
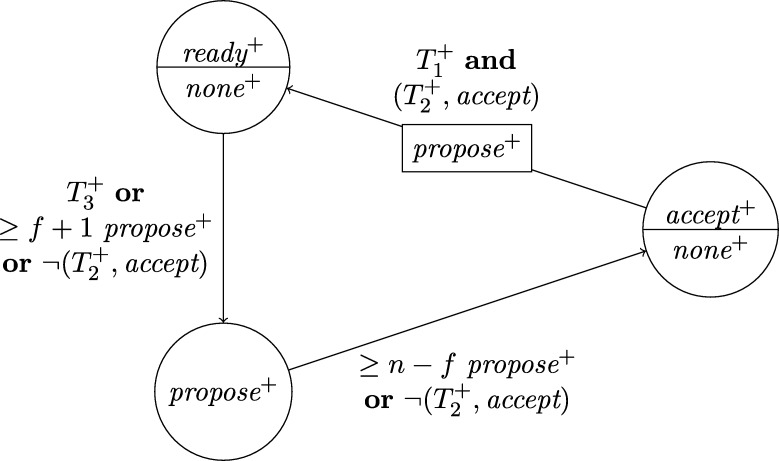
The quick cycle of the FATAL^+^ protocol. Note that we omitted the controls of the logical clock $L_{i}$ that operates modulo $K=2^{b}$, where b∈N is the number of bits of the clock. $L_{i}$ is a simple counter that is increased whenever node *i* switches to state ${\mathrm{accept}}^{+}$ and is reset to 0 when node *i* triggers a pulse, i.e., when the main state machine switches to state *accept*.

• At the top level, we have the *quick cycle* state machine (Fig. 6) that outputs $L_{i}$. The quick cycle is very similar to the algorithm given in Fig. 1, except that it is coupled to the state machine beneath it in order to ensure eventual stabilization. Once the system is stabilized, it consistently and deterministically increases $L_{i}$ at a high frequency while guaranteeing small clock imprecision.
• The *main state machine* (Fig. 8) is the centerpiece of the stabilization mechanism. Once stabilized, it generates slow, roughly synchronized “pulses” within certain frequency bounds. These pulses can be seen as a “heartbeat” of the system; at each pulse, the quick cycle's clocks are reset to 0 and the quick cycle's state machines are forced into state ${\mathrm{accept}}^{+}$ (corresponding to the *increase* state in Fig. 1). This enforces exactly the initial synchrony that we explained to be necessary for the correct operation of the algorithm from Fig. 1.
  By itself, however, the main state machine is not capable of recovering from *every* possible initial configuration of the non-faulty nodes. In certain cases, it requires some coarse synchrony to be established first in order to stabilize, which is probabilistically provided by the underlying layer. We remark that, once stabilized, the main state machine operates fully independently of this layer (and thus deterministically).
• The auxiliary information potentially required for stabilization by the main state machine is provided by a simple intermediate layer we refer to as *extension* of the main state machine (Fig. 9). Essentially, it is supposed to be consistently reset by the underlying layer and then communicate information vital for stabilization to the main state machine. This information depends both on the time of reset and the current states of the *n* main state machines, which it therefore monitors.
• Finally, the *resynchronization routine* (Fig. 10) utilizes randomized timeouts to consistently generate events at all non-faulty nodes that could be understood as “randomized pulses”. Such a pulse is correct for our purposes if all non-faulty nodes generate a respective event in coarse synchrony and no non-faulty node generates another such event within a time window of a certain length. The crux of the matter is that a single such pulse suffices to achieve stabilization deterministically. Relying on (pseudo-)randomness on this layer greatly simplifies the task of overcoming the interference by faulty nodes at low costs in both time and communication. We note that the main state machine masks this randomness once stabilization is achieved, facilitating deterministic behavior of the higher levels and, ultimately, the nodes' clocks $L_{i}$.

We will now present the individual state machines. We refrain from a discussion of choosing appropriate durations for the timers, confining ourselves to stating a feasible family of choices later on.

### 5.3 The quick cycle

The quick cycle state machine is depicted in Fig. 6. It introduces an additional notation: As the states ${\mathrm{ready}}^{+}$ and ${\mathrm{accept}}^{+}$ are not distinguished in any of the transition conditions in the other state machines, the same state ${\mathrm{none}}^{+}$ can be communicated here. This allows for a very efficient single-bit representation of the communicated states. In Fig. 6, this is expressed by dividing the circles representing states, putting the state names in the upper part and the communicated states in the lower part. Apart from saving a wire, this permits to use trivial encoding and decoding of the signal, a simplification of the logic that minimizes delays and therefore maximizes the clock frequency that can be achieved.

Essentially, the quick cycle works as the algorithm given in Fig. 1, where the logical clock is increased whenever the machine switches to state ${\mathrm{accept}}^{+}$. However, the quick cycle differs from the algorithm in Fig. 1 in that there is an interface to the main state machine given in Fig. 8. These state machines communicate by means of two signals only, one for each direction of the communication: (i) The quick cycle state machine of node *i* generates the ${\text{next}}_{i}$ signal by which it exerts some limited influence on the time between two successive pulses generated by the main state machine, and (ii) it observes the $\left(T_{2}^{+},\mathrm{accept}\right)$ timer. This timer is coupled to the state *accept* of Fig. 8, in which the pulse synchronization algorithm generates a new pulse. The signal's purpose is to enforce a consistent reset of the quick cycle state machine (once the main state machine has stabilized). The feedback mechanism (i) makes sure that, during regular operation, the reset of the quick cycle does not have any effect on the clocks. This is guaranteed by triggering pulses (by means of the non-faulty nodes briefly changing the ${\text{next}}_{i}$ signal to 1 and back to 0 again) exactly at the wrap-around of the logical clock $L_{i}$, i.e., at the time when $L_{i}$ is “increased” from the maximal clock value $K-1=2^{b}-1$ (of a *b*-bit clock) to 0=KmodK.

Similar to Fig. 1, the transition conditions of the quick cycle ensure that the logical clocks never have a clock imprecision of more than one. To increase the frequency further, each node could increase the number of clock “ticks” generated in each iteration of the quick cycle by means of a high-frequency local clock (essentially, a watchdog timer together with a counter), at the expense of larger clock imprecision (see [13]).

### 5.4 Main state machine

**Fig. 7:**
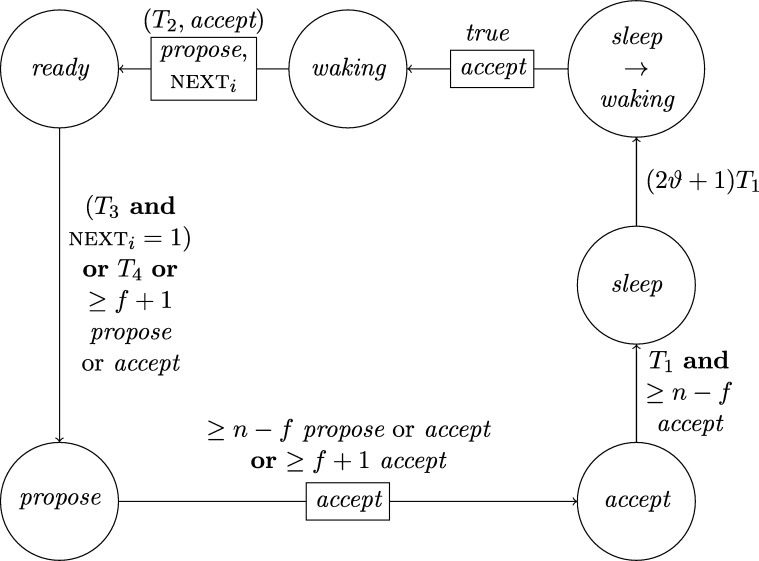
Basic cycle of node *i* once the algorithm has stabilized.

Before we show the complete main state machine, consider its *basic cycle* depicted in Fig. 7. Once the main state machines have stabilized, all non-faulty nodes will undergo the states of the basic cycle in rough synchrony. The states *sleep*, sleep→waking, and *waking* serve diagnostic purposes related to the stabilization process. The duration $T_{2}$ of the timer $\left(T_{2},\mathrm{accept}\right)$ triggering the transition from *waking* to *ready* is so large that the node will always be in state *waking* long before the timer expires. Thus, we can see that the basic cycle has an underlying structure that is very similar to the quick cycle. Due to the more complicated logic and conditions on the duration of timers required for the stabilization mechanism, it is however executed at a frequency that is by orders of magnitude smaller than that of the quick cycle.

The difference in the rules for switching to *propose* and *accept*, respectively, are also mostly related to the stabilization process. An exception is the condition “$T_{3}$ **and** ${\text{next}}_{i}=1$” that can trigger a transition from *ready* to *propose*. Choosing $T_{3}$ smaller than $T_{4}$ and taking the signal ${\text{next}}_{i}$ into account, we permit the quick cycle to adjust the time between pulses (i.e., switches to *accept*) triggered by the main state machine: Once both state machines are roughly synchronized among all non-faulty nodes, the main state machines will always be in state *ready* before the logical clocks $L_{i}$ maintained by the quick cycle reach the wrap-around (i.e., become 0 modulo *K*) and trigger the ${\text{next}}_{i}$ signals. Moreover, this happens at all nodes at close times and before any timer $\left(T_{4},\mathrm{ready}\right)$ expires at one of the non-faulty nodes. Hence, by a reasoning similar as for Fig. 1, all non-faulty nodes will switch to *propose* and subsequently *accept* in a well-synchronized fashion, caused by the wrap-around of the logical clocks.

An important observation that is proved in [13] is that, once the main state machines stabilized, the nodes execute the basic cycle deterministically and any state transition is certainly completed *before* one of the conditions for leaving the basic cycle can be satisfied. Apart from small additional slacks in the timer durations, this is a consequence of the fact that none of the transition conditions of the basic cycle refer to the probabilistic lower layers of the protocol; all evaluated timers and memory flags solely involve states of the basic cycle only, and the ${\text{next}}_{i}$ signal is provided by the quick cycle. As we will discuss in Section 6, this property prevents non-faulty nodes from introducing metastability once stabilization is achieved.

**Fig. 8:**
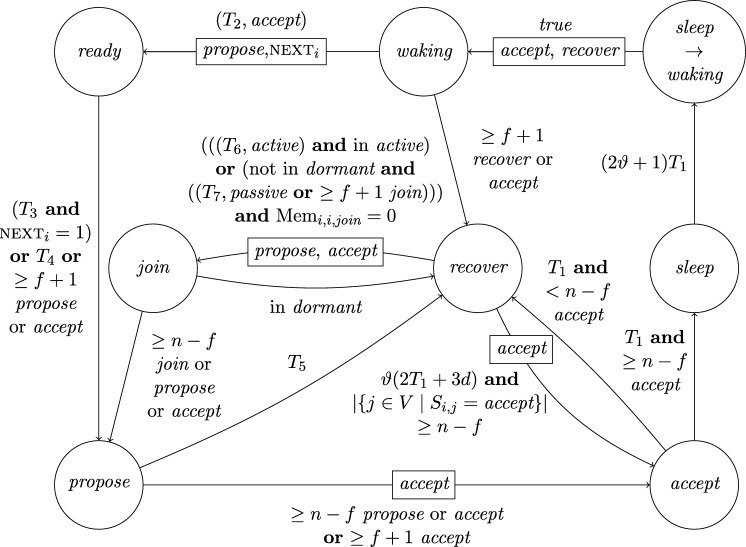
Overview of the main state machine of node *i*.

We now turn our attention to the full main state machine that is shown in Fig. 8. Compared to the basic cycle, we have two additional states, *resync* and *join*, that can be occupied by non-faulty nodes during the stabilization process only, and an additional reset of memory flags on the transition from sleep→waking to *waking*.

The various conditions for leaving the basic cycle and switching to *recover* are consistency checks. A node will only leave the basic cycle if it is certain that the system is not operating as desired. As the high-level operation of the algorithm is not the subject of this article, we limit our exposition to briefly discussing the two possible ways to re-enter the basic cycle, corresponding to two different stabilization mechanisms.

The first stabilization mechanism is very simple, and it is much faster than the second one. Assuming that at least n−f non-faulty nodes are executing the basic cycle (i.e., the main state machines have already stabilized if we consider the remaining nodes faulty), a recovering node just needs to “jump on the train” and start executing the basic cycle as well. This is realized by the condition for switching from *recover* to *accept*. It is not hard to see that due to this condition, the node will switch to *accept* in sufficient synchrony with the majority of n−f synchronized, non-faulty nodes within at most two consecutive pulses and subsequently follow the basic cycle as well.

Note that this condition makes direct use of the state signals instead of using memory flags. This potentially induces metastability at the joining node, but we will explain in Section 6 why the risk is low.^17^ On the plus side, this simplifies the algorithm, as the node does not need to implement frequent resets of the respective memory flags to ensure consistent observation of others' states; the sending nodes will just do this implicitly by leaving state *accept*.

Clearly, the first stabilization mechanism will fail in certain settings. Most obviously, it cannot “restart” the system if all nodes are in state *recover*. Hence it may not surprise that the second stabilization mechanism, which deals with such cases, is much more involved. Careful attention has to be paid to avoiding the potential for system-wide dead- or live-locks. In view of our design goals, state-of-the-art deterministic solutions for this problem are not sufficiently efficient. Hence, the main state machine relies on a probabilistic lower layer that provides certain guarantees with a very large probability.

### 5.5 Extension of the main state machine

**Fig. 9:**
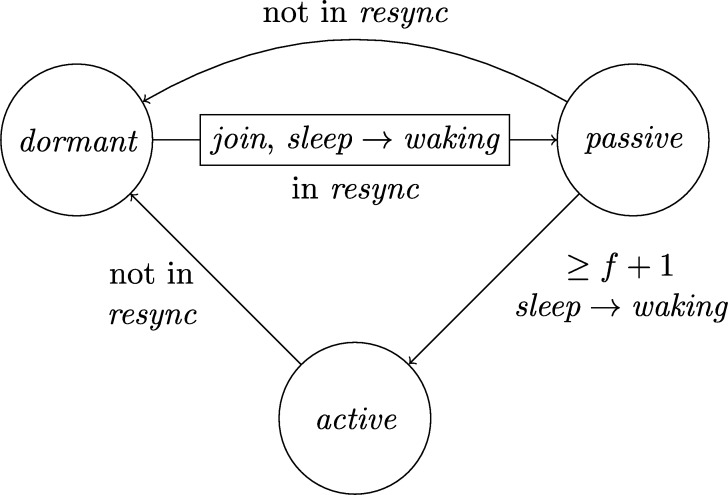
Extension of node *i*'s core routine. Note that the state transition module has access to the full state of node *i*'s resynchronization state machine, even though *i* communicates less information to remote nodes *j* ≠ *i* (see Section 5.6). Hence the conditions “in *resync*” and “not in *resync*” can be evaluated.

The extension of the main state machine, given in Fig. 9, can be seen as a simple control structure for the phases of stabilization. The intricacy lies in designing the interface such that this control does not interfere with the basic cycle if the system is stable. Consequently, the influence of the extension of the main state machine is limited to (i) resetting the *join* and sleep→waking flags upon “initializing” the stabilization process (by switching from *dormant* to *passive*) and (ii) providing the signals of the timers $\left(T_{6},\mathrm{active}\right)$ and $\left(T_{7},\mathrm{passive}\right)$ the main state machine utilizes in the transition rule from *recover* to *join*.

Roughly speaking, the main state machines will stabilize deterministically under the condition that their extensions switch at all non-faulty nodes from *dormant* to *passive* in rough synchrony and then do not switch back to *dormant* too quickly, i.e., before the second stabilization mechanism of the main state machine completes its work. Putting it simply, we require a single, coarsely synchronized pulse, whose generation is the purpose of the lowest layer we present now.

### 5.6 Resynchronization state machine

**Fig. 10:**
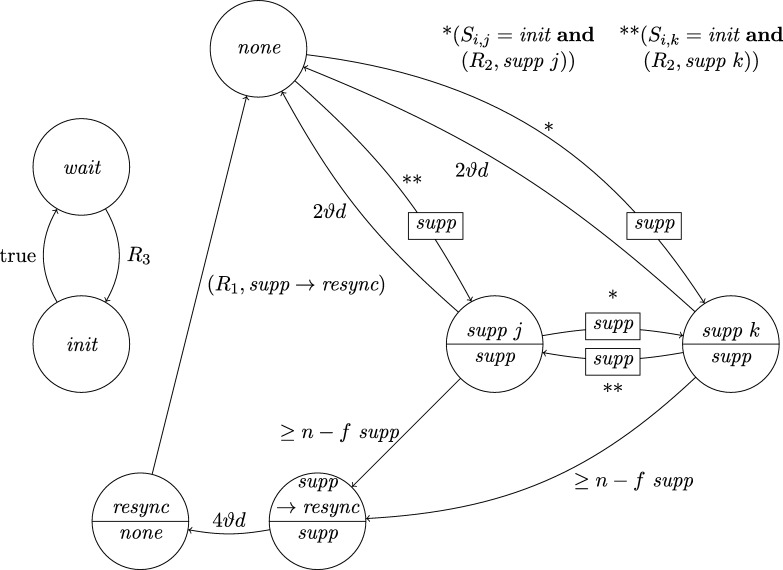
Resynchronization state machine at node *i*. The states *supp* *j* and *supp* *k* are representative for *n* states *supp* 1,…,*supp* *n* corresponding to the *n* nodes of the system; all these states are built in the same way with symmetric transition conditions.

The resynchronization state machine is specified in Fig. 10. Strictly speaking, it actually consists of two separate state machines, one of which is however extremely simple. Every now and then, each node will briefly switch to the *init* state, seeking to induce the generation of a “pulse” (where the pulse here is locally triggered by switching to *resync*) that causes a consistent switch of all non-faulty nodes from *dormant* to *passive*. Leaving *resync* will force the extension state machine back into state *dormant*. This is the only interaction with the above layer, which is sufficient if a pulse is successfully generated once.

The generation of a pulse is achieved by all non-faulty nodes following the advice of a *single* node switching to *init*, thus establishing the common time base required for a synchronized pulse. Two obstacles are to be overcome: possibly some of the non-faulty nodes already believe that the system is in the middle of an attempt to stabilize (i.e., they are already in state *resync* and thus not ready to follow the advice given by another node) and possibly inconsistent information by nodes that remain faulty (causing only *some* of the non-faulty nodes to switch to *resync*).

In contrast to the higher levels, however, we are satisfied if only *occasionally* a successful pulse is generated. Hence, the above issues can be overcome by randomization. The source of randomness here is the randomized timer $\left(R_{3},\mathrm{wait}\right)$. The distribution $R_{3}$ and the logic of the second, more complicated state machine including the state *resync* are designed such that there is a large probability that within time O(n) all non-faulty nodes will consistently switch to state *resync*. This O(n) is essentially the factor by which the second stabilization mechanism of the main state machine is slower than the first one.

### 5.7 Timer durations

Clearly, in order for the protocol to operate as desired, the timer durations need to satisfy certain constraints. We state a feasible family of durations here; the minimal constraints that are required by the proofs are given in [13].

Recall that ϑ>1 and that *d* bounds the maximal end-to-end delay incurred between the time when a state transition condition is met and the time when the respective signal transition is observed at all receivers. As the logic of the quick cycle is much simpler than that of the other state machines, it typically permits much tighter upper and lower bounds on this end-to-end delay. As in [13], these bounds are denoted by $d_{\min}^{+}$ and $d_{\max}^{+}⩽d$. In Section 6, we will discuss how *d*, $d_{\min}^{+}$, and $d_{\max}^{+}$ can be computed out of the constituent delays incurred in our basic modules.

Defining

$$\lambda :=\sqrt{\left(25\vartheta -9)/(25\vartheta \right)}\in (4/5,1)\;\text{and}\;\alpha :=(T_{2}+T_{4})/\left(\vartheta (T_{2}+T_{3}+4d)\right),$$

for any ϑ>1, α⩾1, the following family of timeout durations meets the requirements stated in [13] (see the reference for a proof):

$$T_{1}^{+}:=6\vartheta^{2}d+6\vartheta^{2}d_{\max}^{+}-\vartheta d_{\min}^{+}$$

$$T_{2}^{+}:=3\vartheta d+3\vartheta d_{\max}^{+}$$

$$T_{3}^{+}:=6\vartheta^{3}d+6\vartheta^{3}d_{\max}^{+}-\vartheta^{2}d_{\min}^{+}$$

$$T_{1}:=4\vartheta d$$

$$T_{2}:=46\vartheta^{3}d/(1-\lambda )$$

$$T_{3}:=\left(\vartheta^{2}-1\right)46\vartheta^{3}d/(1-\lambda )+31\vartheta^{3}d$$

$$T_{4}:=46\vartheta^{3}\left(\alpha \vartheta^{3}-1\right)d/(1-\lambda )+35\alpha \vartheta^{4}d$$

$$T_{5}:=46\vartheta^{4}\left(\alpha \vartheta^{3}-1\right)d/(1-\lambda )+39\alpha \vartheta^{5}d$$

$$T_{6}:=46\vartheta^{4}d/(1-\lambda )$$

$$T_{7}:=92\alpha \vartheta^{8}d/(1-\lambda )+78\alpha \vartheta^{5}d$$

and further,

$$R_{1}:=46\vartheta^{6}\left(3\alpha \vartheta^{3}-1\right)d/(1-\lambda )+109\alpha \vartheta^{6}d$$

$$R_{2}:=\left(92\vartheta^{7}\left(3\alpha \vartheta^{3}-1\right)/{\left(1-\lambda \right)}^{2}+\left(218\alpha \vartheta^{7}+108\vartheta^{3}\right)/(1-\lambda )\right)(n-f)d$$

$$R_{3}:=\text{uniformly distributed random variable on}\;\left[3\vartheta d+\left(92\vartheta^{8}\left(3\alpha \vartheta^{3}-1\right)/{\left(1-\lambda \right)}^{2}+\left(218\alpha \vartheta^{8}+108\vartheta^{4}\right)/(1-\lambda )\right)(n-f)d,3\vartheta d+\left(8(1-\lambda )+\vartheta \right)\left(92\vartheta^{7}\left(3\alpha \vartheta^{3}-1\right)/{\left(1-\lambda \right)}^{2}+\left(218\alpha \vartheta^{7}+108\vartheta^{3}\right)/(1-\lambda )\right)(n-f)d\right].$$

Finally, the maximal logical clock value K−1 is not arbitary, as we require

(1) $$K\in \left[\left(46\vartheta^{4}/(1-\lambda )+52\vartheta^{2}\right)/\left(12+10d_{\max}^{+}/d\right),\alpha \left(46\vartheta^{4}/(1-\lambda )+32\vartheta^{2}\right)/\left(12+12d_{\max}^{+}/d\right)\right].$$

Note that, by manipulating *α*, we can make *K* arbitrarily large, but this comes at the expense of a proportional increase in the timer durations of the main state machine and its underlying layers, increasing the overall stabilization time.

### 5.8 Summary of results from theory

We conclude the section with a summary of the most important statements proved in [13], expressed in terms of the model employed in this article. To this end, we need to specify the protocol as a compound implementation about that we will formulate our theorems.

Definition 5.1 **The FATAL^+^ Protocol**

The *FATAL*^+^ *protocol* is a compound module consisting of nodes i∈{1,…,n}. It has no input ports and an output port $L_{i}$ for each node *i*. The *n* input ports of node *i* are connected to the output ports of the channels $S_{i,j}$, j∈{1,…,n}. Each node is comprised of one copy of each of the state machines presented in this section, and the implementation of each node is derived from the implementations (given in Section 6) of the basic modules defined in Section 2.2 that are connected as specified in this section. The output port $L_{i}$ of node *i* is the output port of its quick cycle state machine.

The first theorem states a probabilistic stabilization result. Since we did not formally define probabilistically stabilizing implementations, its formulation is somewhat cumbersome. Intuitively (and slightly inaccurately), the statement is to be read as “no matter what the initial state and the execution, the protocol stabilizes almost certainly within $T_{\mathrm{slow}}$ time”.

Theorem 5.2 *Fix any* $f^{\prime }⩽f:=\lfloor (n-1)/3\rfloor$ *and feasible α, set*

$$T_{\mathrm{slow}}:=\left(24(1-\lambda )+3\vartheta \right)R_{2}+R_{1}/\vartheta +T_{1}^{+}+T_{3}^{+}+(9\vartheta +8)d+5d_{\max}^{+}-d_{\max}^{-}\in \Theta (\alpha n),$$

*and pick* K∈Θ(αn) *in accordance with inequality* (1)*.*

*Consider an execution on* $\left[t^{-},t^{+}\right]$ *of the FATAL*^+^ *protocol where* (*at least*) $n-f^{\prime }$ *nodes are feasible. Assume that an adversary that knows everything about the system except that it does not learn about the durations of randomized watchdog timers before they expire controls all other aspects of the execution* (*clock drifts and delays of feasible submodules within the admissible bounds as well as the output ports' signals of faulty modules*)*. Then the execution restricted to* $\left[t^{-}+T_{\mathrm{slow}},t^{+}\right]$ *is with probability at least* $1-2^{-(n-f)}$ *a feasible execution of a clock synchronization module with clock imprecision* Σ=1*, amortized frequency bounds* $A^{-}=1/(T_{1}^{+}+T_{3}^{+}+3d_{\max}^{+})$ *and* $A^{+}=1/(\vartheta (T_{1}^{+}+T_{3}^{+}))$*, slacks* $\tau^{-}=\tau^{+}=2$*, maximum frequency* $F^{+}=1/(\vartheta (T_{1}^{+}+T_{3}^{+}-2d_{\max}^{+}+d_{\min}^{+}))$*, at most* $f^{\prime }$ *faults, and clocks of size* K∈Θ(αn)*.*

In this sense, for each $f^{\prime }⩽f$, the FATAL^+^ protocol is an $f^{\prime }$-tolerant implementation of a clock synchronization module with the respective parameters that stabilizes with probability at least $1-2^{-(n-f)}$ within time $T_{\mathrm{slow}}\in O(\alpha n)$.

The above theorem corresponds to the slow, but robust, second stabilization mechanism. The next theorem, which corresponds to the faster first stabilization mechanism, essentially states that in an execution where n−f nodes already stabilized, any further non-faulty nodes recover quickly and deterministically, within O(α) time.

Theorem 5.3 *We use the notation of the previous theorem. Moreover,*

$$T_{\mathrm{fast}}:=T_{2}+T_{4}+\left(1+5/(2\vartheta )\right)R_{1}+5d\in \Theta (\alpha ).$$

*Suppose an execution of the FATAL*^+^ *protocol is feasible on* $\left[t^{-},t^{+}\right]$ *with respect to the clock synchronization module specified in* Theorem 5.2*. Consider the set of nodes* W⊆N *whose restricted executions on* $\left[t^{-},t^{+}\right]$ *are feasible. Then the execution restricted to* $\left[t^{-}+T_{\mathrm{fast}},t^{+}\right]$ *is feasible with respect to a clock synchronization module with the same parameters, except that it tolerates* n−|W| *faults only.*

We should like to mention that in [13] a number of further results on stabilization are given. In particular, if the faulty nodes exhibit only little coordination among themselves or do not tune their operations to the non-faulty nodes' states, also the “slow” stabilization mechanism will succeed quickly, granted that the resynchronization state machines are not in a “too bad” configuration, i.e., most timers of type $R_{2}$ are expired and timeouts of type $R_{3}$ are in (roughly) random states. We will informally discuss some of these scenarios in Section 7.

Finally, we emphasize again that the power of the above theorems severely depends on the quality of basic implementations (cf. Section 4.2). While compound modules' properties can be formally analyzed, e.g. giving rise to the theorems above, these results are meaningless if too many basic implementations are infeasible too frequently. Hence it is vital to come up with robust implementations of the basic modules, which is the subject of the next section.

## 6 Implementation

In this section, we present the cornerstones of our FPGA prototype implementation of the FATAL^+^ protocol. The objectives of this implementation are (i) to serve as a proof of concept, (ii) to validate the predictions of the theoretical analysis, and (iii) to form a basis for the future development of protocol variants and engineering improvements. Rather than striving for optimizing performance, area, or power efficiency, our primary goal is hence to essentially provide a direct mapping of the algorithmic description to hardware, and to evaluate its properties in various operating scenarios.

Not surprisingly, traditional design principles for digital circuits are not adequate for our purposes. This is true for three major reasons:

• **Asynchrony:** Targeting ultra-reliable clock generation in SoCs, the implementation of FATAL^+^ itself cannot rely on the availability of a synchronous clock. Moreover, many guards, like the one of the transition from *propose* to *accept* in Fig. 8, depend on remote nodes' states and should hence not be synchronized to a local clock in order to maximize performance. Testing for activated guards synchronized to a local clock source also increases the risk of generating metastability, as remote signals originate in different clock domains. On the other hand, conventional *asynchronous state machines* (ASM) are not well-suited for implementing the state machines from Fig. 6–Fig. 10 due to the possibility of choice w.r.t. successor states and continuously enabled (i.e., non-alternating) guards. Our prototype hence relies on *hybrid state machines* (HSM) that combine ASM with synchronous *transition state machines* (TSM) that are started on demand only.
• **Fault tolerance:** The consideration of Byzantine faulty nodes forced us to abandon the classic “wait for all” paradigm traditionally used for enforcing the indication principle in asynchronous designs: Failures may easily inhibit the completion of the request/acknowledge cycles typically used for transition-based flow control. A few timing constraints, established by our theoretical analysis, in conjunction with state-based communication are resorted to in order to establish event ordering and synchronized executions in FATAL^+^.
• **Self-stabilization:** In sharp contrast to non-stabilizing algorithms, which can always assume that there is a (substantial) number of non-faulty nodes that run approximately synchronously and hence jointly adhere to certain timing constraints, self-stabilizing algorithms cannot even assume this. Although FATAL^+^ guarantees that non-faulty nodes will eventually execute synchronously, even when started from an arbitrary state, the violation of timing constraints and hence metastability cannot be avoided during stabilization [44]. For example, state *accept* in Fig. 8 has two successors *sleep* and *recover*, the guards of which could become true arbitrarily close to each other in certain stabilization scenarios. This is acceptable, though, as long as such problematic events are neither systematic nor frequent, which is ensured by the design and implementation of FATAL^+^ (see Section 6.1).

Inspecting Figs. 6–10 reveals that the state transitions of the FATAL^+^ state machines are triggered by AND/OR combinations of the following different types of conditions:

(1) A watchdog timer expires [“$\left(T_{2},\mathrm{accept}\right)$”].
(2) The state machines of a certain number (1, ⩾f+1, or ⩾n−f) of nodes reached a particular (subset of) state(s) at least once since the reset of the corresponding memory flags [“⩾n−f *accept*”].
(3) The state machines of a certain number (1, ⩾f+1, or ⩾n−f) of nodes are currently in (one of) a particular (subset of) state(s) [“in *resync*”].
(4) Always [“true”].

These requirements reveal the need for the following major building blocks (cf. Section 4):

• Concurrent HSMs, implementing the states and transitions specified in the protocol. An ideal HSM would always provide feasible executions of its state transition module.
• Communication infrastructure between these state machines, continuously conveying state information. This is simply done by the channels $S_{i,j}$ propagating the signal $S_{i}$ to all receivers.
• Watchdog timers (also with random timeouts) for implementing type (1) guards.
• Threshold modules and memory flags for implementing type (2) and type (3) guards.

If we could provide implementations of *all* these building blocks that match the specifications of the formal model in Section 2.2 under *all* circumstances, in the sense that all executions at non-faulty nodes are always feasible, the theoretical guarantees derived in [13] would apply without restriction. As already noted, however, this is impossible to guarantee, since there is no way to rule out metastable upsets with complete certainty, and there are no elements available for our purpose whose behavior is specified for metastable inputs. Nevertheless, it is possible to design our basic modules in a way that keeps the probability of such events acceptably low. Moreover, all stateful components must be implemented in a self-stabilizing way: They must be able to eventually recover from an arbitrary erroneous internal state, including metastability, when facing sufficiently long executions on their input ports that do not induce metastability.

Before we proceed with a description of the implementations of the required basic modules, we discuss how FATAL^+^ deals with the threat of metastability arising from our extreme fault scenarios.

### 6.1 Metastability issues

Reducing the potential for both metastability generation and metastability propagation are important goals in the design and implementation of FATAL^+^. Although it is impossible to completely rule out metastability generation in the presence of Byzantine faulty nodes (which may issue signal transitions at arbitrary times anyway) and during self-stabilization (where all nodes may be completely unsynchronized), we nevertheless achieve the following properties.

Robustness against metastable upsets and their propagation:

(I) Guaranteed *metastability-freedom in fault-free executions* after stabilization.
(II) *Low probability of metastable upsets*: We have taken care to keep the windows of vulnerability of our implementations of basic modules as small as possible. Thus, desynchronized or faulty nodes must be very lucky to actually trigger a metastable upset. In addition, mechanisms for decreasing the upset probability even further could be incorporated, if required in particularly critical applications.
(III) *Metastability containment*: Non-faulty nodes are very robust against propagation of metastable upsets due to the algorithm's control flow.

Limited consequences of metastable upsets:

(IV) *Limited impact of metastable upsets during stabilization*: Metastable upsets that occur at non-faulty nodes during the stabilization phase can only delay stabilization. Since these are rare events even then, the respective effect on the (average) stabilization time is very small.
(V) *Fast recovery from metastability after stabilization*: As long as n−f non-faulty nodes remain synchronized, a metastable upset at a node may disrupt its synchrony towards the other nodes only for a short time. Due to the fast stabilization mechanism the node will fully recover within O(1) time once metastability ceases.
(VI) *Masking of metastable upsets as faults*: Provided that the measures ensuring (II) and (III) are effective (i.e., metastability does not spread) and the system-level fault-tolerance of *f* nodes operating outside their specification is not exhausted, metastable upsets at some nodes do not affect the correctness of other nodes.

The following approaches have been used in FATAL^+^ to accomplish these goals (additional details will be given in the subsequent sections):

(I) If all nodes are synchronized and fault-free, we can satisfy timing constraints on the modules' input ports' signals that ensure that even our (necessarily imperfect) implementations of the abstract modules maintain feasibility at all times. Essentially, the argument is that since there is no initial violation of the constraints and no faults are imposed by external events, we can conclude that the constraints will be satisfied at later points in time as well. This property is formally proved in [13].
(II) All building blocks that are susceptible to metastable upsets, like memory flags, are implemented in a way that minimizes the time span during which they are vulnerable. Moreover, elastic pipelines acting as metastability filters [40] or synchronizers could be added easily to our design to further protect such elements.
(III) We enforce (standard) error containment by avoiding any explicit control flow between ASMs: Since the communication is exclusively performed by virtue of states, a faulty receiver cannot impact a non-faulty sender, and a faulty sender, in turn, cannot directly interfere with the operation of a non-faulty receiver (apart from conveying an incorrect state, of course). To extend error containment to also cover metastability to the best possible extent, several forms of logical masking are employed. One example is the combination of memory flags and threshold gates, which ensure that possibly upset memory flags are always overruled quickly by correct ones at the threshold output.^18^ A higher-level form of logical masking occurs due to the fact that, after stabilization, all non-faulty nodes execute the outer cycle of the main state machine (Fig. 8) only. The outer cycle's guards do not involve any of the timeouts, states, or flags accessed by the resynchronization routine (Fig. 10) or the extension of the main state machine (Fig. 9); hence any metastability of the corresponding signals does not affect the logic of the main state machine and the layers on top of it (including the logical clocks).
(IV) The measures outlined in (II) and (III) are complemented by adding time masking using randomization. The resynchronization routine (Fig. 10) tries to initialize recovery from arbitrary states at random, sufficiently sparse points in time. Hence non-faulty nodes cannot be systematically kept from stabilizing. The proofs in [13] reveal that within O(n) time in fact it is likely that there are multiple events that will imply subsequent stabilization. Considering that metastable upsets are rare events in our setting, their impact thus becomes negligible.
(V) This property directly follows from the results shown in [13]: If n−f nodes faithfully execute the basic cycle, any non-faulty node will (re)synchronize within O(1) time, irrespectively of its current state.
(VI) If metastability does not spread to a given receiver, the latter will observe for each sender some execution, even if the sender does not send a valid signal in terms of our system model. Since we assume that faulty nodes may output arbitrary signals at their output ports, our model thus makes no distinction between a “conventionally” faulty node and one that behaves erratically due to metastable upsets.^19^ As the algorithm is resilient to up to *f* faults, such upsets are masked as long as the total number of nodes operating outside their module specification is at most *f*.

### 6.2 State machine communication

According to our system model, an HSM of node *i* must continuously communicate its current state system-wide via the channels $S_{j,i}$. For simplicity, we use parallel communication, by means of a suitably sized data bus, in our implementation.^20^ A complete receiver as described below is employed for every state machine in the system. Since a node treats itself like any other node in type (2) and type (3) guards with thresholds, every node receives its own state as well.

#### 6.2.1 Channels

**Fig. 11:**
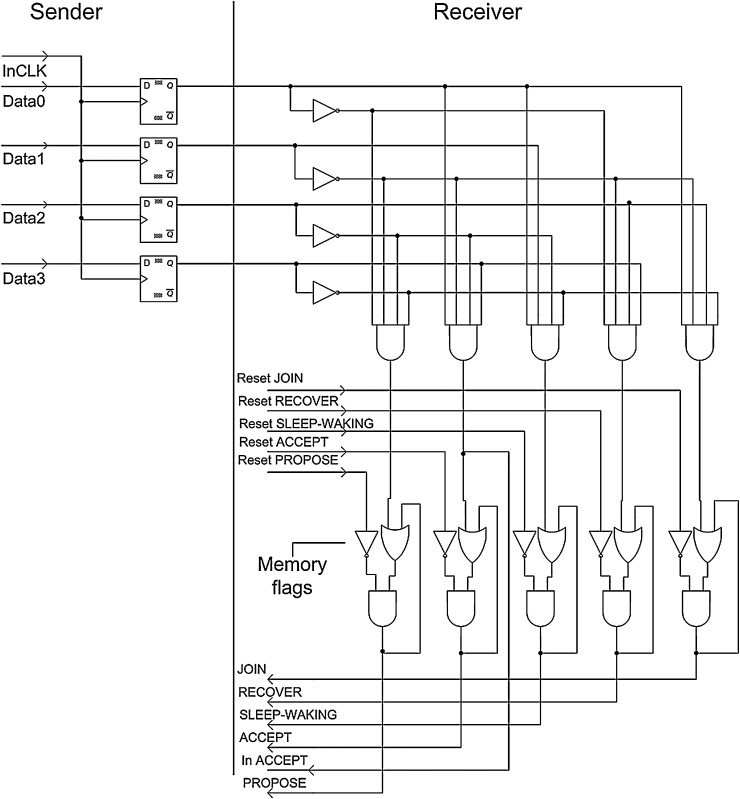
Sender and single receiver (including memory flags) for the ASM of the main algorithm.

Fig. 11 shows the circuitry used for communicating the current state of the main algorithm in Fig. 8.

The sender consists of a simple array of flip-flops, which drive the parallel data bus that thus continuously reflects the current state of the sender's HSM. Technically speaking, the flip-flops are not part of the channel but rather the sender's HSM; they are the “physical location” of the HSM's state in the sense of our model. The channel thus “begins” with the wires conveying the stored values.^21^

In sharp contrast to handshake-based communication, reading at the receiver occurs without any direct coordination with the sender. To avoid the unacceptable risk of reading and capturing false intermediate sender states, which might be perceived by the receiver upon a sender state transition in case of different delays on the data bus wires, delay-insensitive state coding [45] must be used. We have chosen the following encoding for the main state machine in Fig. 8:

**Table:** 

*propose*	0000	*accept*	1001
*sleep*	1011	*sleep* → *waking*	0011
*waking*	0101	*ready*	0110
*recover*	1100	*join*	1010

The receiver comprises a simple combinational decoder consisting of AND gates, which generate a 1-out-of-*m* encoding of the binary representation of the state communicated via the data bus. The decoded signals correspond to a single sender state each. This information is directly used for type (3) guards, and fed into memory flags for type (2) guards.

For the other state machines making up FATAL^+^, it suffices to communicate only a single bit of state information (*supp* or *none* in Fig. 9, *init* or *wait* in Fig. 10, and ${\mathrm{propose}}^{+}$ or *none*^+^ in Fig. 6). Hence, every bus consists of a single sender flip-flop plus a wire here, and the decoder in the receiver becomes trivial. In the sequel, we restrict our discussion to the main state machine's channel, as the simpler single-bit channels clearly meet the specification of a channel. Note that in both cases the (physical) channels used in our implementation trivially recover from any inputs and transient faults, as they are obviously forgetful. The memory flags at the receiver's side contain feedback-loops, however, which do not allow us to apply Theorem 3.7 and Lemma 3.8.

##### Correctness.

We now argue informally^22^ why and when the above implementation matches the specifications given in Section 4. Note that when affected by faults or provided with illegal inputs, modules may of course exhibit arbitrary behavior. In that case we rely on (a) the system-level fault tolerance properties (for fault masking), (b) the self-stabilization properties of the affected modules (for recovery), and (c) the rare occurrence of these situations (in order to not exhaust the system-level fault tolerance limits). In addition to considering the fault-free behavior, it hence suffices to restrict our attention to (b) and (c) here.

For fault-free operation, the described implementation essentially realizes a channel as specified in Section 4 with some maximum delay $d_{\mathrm{Chan}}$, granted that changes of the input provided by the sender are separated in time sufficiently well. To see this, consider an input switch from state *s* to $s^{\prime }$ (note that not all flip-flops will switch their output signals at exactly the same instant), where initially the signal is stable also on the receiver's side. Once the signal change propagated through the wires and the AND gates, the decoder output signal corresponding to state $s^{\prime }$ will be 1, while all other signals will be 0. Due to the use of delay-insensitive state encoding, there are no glitches and the signals for all other states $s^{\prime \prime }\notin \{s,s^{\prime }\}$ will continuously be 0. Nevertheless, formally, this behavior does not yet fully match the definition of our communication channels in Section 4: It is possible that temporarily both *s* and $s^{\prime }$ are 1. Since our algorithms are completely oblivious to the exact point in time when the perceived $S_{i,j}$ changes after the sender's state $S_{j}$ changed (the analysis in [13] only requires that this happens within *d* time), however, this problem can easily be abstracted away.^23^ All that is needed here is to interpret, in a static way, the situation where both *s* and $s^{\prime }$ are valid as, say, *s*.

The attentive reader will have noticed that the 1-out-of-*m* decoder outputs (i.e., the state signals at the inputs of the memory flags) may temporarily be all 0 during the reception of a sender state transition as well. Fortunately, this behavior is completely masked from becoming visible to our algorithms: The memory flags prohibit this from becoming visible in type (2) guards at all, and all state transition conditions involving type (3) guards refer to a single state only. Hence, in terms of the transition condition, a similar abstraction as above is valid (i.e., for a remote state transition from *s* to $s^{\prime }$ with a “gap” we can define an equivalent execution without gap in which the node in question behaves identically).

The above arguments critically rely on the assumption that states change not too rapidly. Otherwise, the receiver could e.g. fail to observe states that the sender assumed for a too short period of time only, or even decode a state that has not been attained. For non-faulty nodes, this is guaranteed in our implementation because the minimal amount of time an HSM needs to complete a state transition is greater than the maximum end-to-end delay variation of the signals employed in the communication channel. This constraint is easy to ensure by proper circuit design rules.

##### Metastability.

Within the communication channels themselves, metastable upsets could only occur in the senders' flip-flops and in the receivers' memory flags; everything else is stateless combinational logic. The flip-flops are clocked by the sender's own clock, hence could become metastable only in case of a faulty sender. The issue of upsets of the memory flags is discussed in Section 6.2.2.

Viewed at the node level, it is obvious that if the sender's state signal becomes metastable or changes too quickly (which can only happen if the sender is faulty), this can also induce metastability at the receiver side by propagation over the channel. During the stabilization phase, the receiver could also experience a channel-induced metastable upset in memory flags and/or in its HSMs due to the arbitrary desynchronization between sender and receiver; since the windows of vulnerability are very small, the upset probability is very low, though. Eventually, after stabilization, the synchrony between non-faulty nodes guaranteed by the FATAL^+^ protocol ensures that the received state data will always be stable when read in a transition condition in the main algorithm's outer cycle, recall item (I) in Section 6.1.

#### 6.2.2 Memory flags

Every memory flag is just an SR-latch with dominant reset, whose functional equivalents are also depicted in Fig. 11. Note that a memory flag is set depending on the state communicated by the sender, but (dominantly) cleared under the receiver's control.

##### Metastability.

A memory flag may become metastable when the inputs change during stabilization of its feedback loop, which can occur due to (a) input glitches and/or (b) simultaneous falling transitions on both inputs. However, for correct receivers, (a) can only occur in case of a faulty sender, and (b) is again only possible during stabilization: Once non-faulty nodes execute the outer cycle of Fig. 8, it is guaranteed that e.g. all non-faulty nodes enter *accept* before the first one leaves. Overall, the upset probability is thus very small. It could be further reduced by diverse known means for metastability filtering, like using an elastic pipeline or Schmitt-trigger stages (which must be accounted for in the delay bounds, though). Finally, it is well-known that a metastable flip-flop will recover in finite time with probability one [44].

Any SR latch matches the specification of a memory flag according to Section 4 followed by a channel with some maximum delay $d_{\mathrm{Mem}}$, provided that it starts from a clean initial state and the set/reset signals avoid (a) and (b) above. As argued above, the latter is guaranteed by our algorithm except in case of a metastable upset. In case of the memory flag implementation shown in Fig. 11, $d_{\mathrm{Mem}}$ is primarily determined by the end-to-end settling time of the feedback loop. This delay also determines the vulnerability window with respect to metastability (i.e. critical glitch length, and “simultaneity” of transitions). Hence, making $d_{\mathrm{Mem}}$ small, which is easy to achieve by design, contributes to both speed and robustness.

Except in case of metastability, discussed before, our memory flag implementation is self-stabilizing since it is $d_{\mathrm{Mem}}$-forgetful in the presence of input executions that avoid (a) and (b).

#### 6.2.3 Threshold modules

The most straightforward implementation of the threshold modules used for generating the ⩾f+1 and ⩾n−f thresholds in type (2) and type (3) guards is a simple sum-of-product network, which just builds the OR of all AND combinations of f+1 respectively n−f inputs. This implementation however quickly becomes highly expensive, as it requires $\Theta (\left(\begin{array}{c} n\\ f \end{array}\right))$ gates. A more efficient alternative is a sorting network, where the $k^{th}$ output indicates whether a threshold of *k* is reached. For simplicity, in our FPGA implementation, threshold modules are built by means of lookup-tables (LUT).

##### Correctness.

Similar to our memory flag implementation, it is impossible to implement the properties of a threshold module as stated in Section 4, followed by a channel with some maximum delay $d_{\mathrm{Th}}$, in case of arbitrary inputs: Finding out whether a certain number of inputs is 1 exactly at the same time cannot be implemented with real circuits. All implementations proposed above are forgetful and their outputs will stabilize quickly if their inputs do not change. Moreover, after stabilization type (2) guards are irrelevant, since neither the basic cycle of the main state machine nor the quick cycle evaluate such guards. Hence, in this case we can restrict our attention to input executions where inputs may change from 0 to 1 only, not back. The reset of the memory flags to 0 is performed during state transitions (when the guards' signals are suppressed by the locked signal) and therefore safe.

As any of the proposed threshold module implementations involve combinational logic only, they are trivially self-stabilizing: According to Theorem 3.7, they are forgetful and hence, by Lemma 3.8, self-stabilizing. Therefore, provided that the longest path delay does not exceed $d_{\mathrm{Th}}$, the properties stated in Section 4 are satisfied for monotonic inputs.^24^

##### Metastability.

As discussed above, type (2) guards cannot be safely evaluated by threshold gates and may cause glitches or metastable upsets. Since this is of relevance before stabilization only, this risk is considered acceptable. Like our channel implementations, threshold modules can propagate metastability: A metastable input could be propagated to the output when there are exactly k−1 non-faulty inputs in state 1 and the metastable input therefore makes the difference between output 0 and 1. In all other cases, however, the metastable input will simply be masked. Thus, albeit not perfect, threshold gates are an efficient means for metastability containment.

### 6.3 Hybrid state machines

Our prototype implementation of FATAL^+^ relies on *hybrid state machines* (HSM): An asynchronous state machine (ASM) is used for determining, by asynchronously evaluating the guards, the points in time when a state transition shall occur. Our ASMs have been built by deriving a *state transition graph* (STG) specification directly from Figs. 6–10 and generating the delay-insensitive implementation via Petrify [46]. The actual state transition of an HSM is governed by an underlying synchronous *transition state machine* (TSM). The TSM resolves a possibly non-deterministic choice of the successor state and then sequentially performs the required transition actions:

1. “Locking” the transition, i.e., disabling any other transitions of the ASM (despite possibly satisfied guards); this happens at the start of the TSM and is thus asynchronously triggered.
2. Reset of memory flags and watchdog timers.
3. Communication of the new state, i.e., writing its representation into the flip-flops whose output is fed into the channels $S_{j,i}$.
4. Completing the transition to the new state by enabling further transitions of the ASM.

The TSM is driven by a pausable clock (see Section 6.4), which is started dynamically by the ASM upon triggering the transition. Note that this avoids the need for synchronization with a free-running clock and hence preserves the ASM's continuous time scale.

**Fig. 12:**
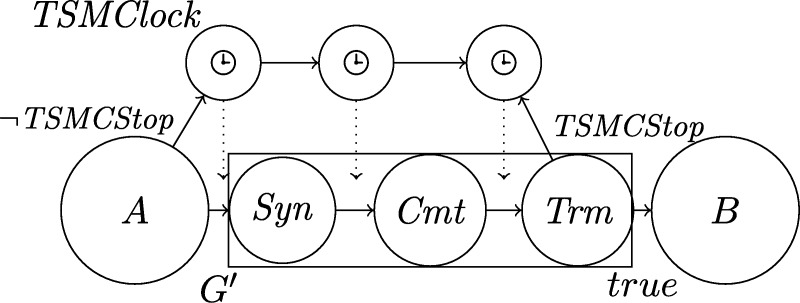
Example state transition, including the corresponding TSM.

The TSM works as follows (see Fig. 12): Assume that the ASM is in state *A*, and that the guard *G* for the transition from *A* to *B* becomes true. If no other transition is currently being taken (indicated by the locked signal being 0), the TSM clock is started and the TSM sequence counter is released. With every rising edge of *TSMClock*, the TSM moves through a sequence of three states: *synchronize* (*Syn*), *commit* (*Cmt*), and *terminate* (*Trm*) shown in the rectangular box in Fig. 12. In *Syn*, the locked signal is activated to prevent other choices from being executed in case of more than one guard becoming true.

Once the TSM has reached *Syn*, it has decided to actually take the transition to *B* and hence moves on to state *Cmt*. Here the watchdog timer associated with *B* and possibly some memory flags are cleared according to the FATAL^+^ state machine, and the new state *B* is captured by the output flip-flops driving the state communication data bus (recall Section 6.2). Note that the resulting delay must be accounted for in the communication delay bounds *d*, $d_{\max}^{+}$ and $d_{\min}^{+}$. Finally, the TSM moves on to state *Trm*, in which the reset signals are inactivated again and the TSM clock is halted (and the TSM sequence counter forced to the reset state). The locked signal is also cleared here, which effectively moves the ASM to state *B*. It is only now that guards pertaining to state *B* may become true.

##### Metastability.

Whereas any ambiguity of state transitions due to multiple activated guards can easily be resolved via some priority rule, metastability due to (a) enabled guards that become immediately disabled again or (b) new guards that are enabled close to “locking” time cannot be ruled out in general. However, as argued in Section 6.1, in FATAL^+^ (a) could only occur during stabilization, due to type (3) guards, or due to faulty nodes successfully inducing metastability of memory flags; recall that otherwise type (1) and type (2) guards are always monotonic, with the reset (of watchdog timers and memory flags) being under the control of the local state machine. Similarly, our proofs in [13] reveal that upsets due to (b) do not occur after stabilization in the main state machine and the quick cycle (Figs. 6 and 8). As the main state machine is logically independent of the lower layers (Figs. 9 and 10) after stabilization, any metastability in these layers is fully masked. Thus, after stabilization, metastability of the TSMs we care about can only occur due to unstable inputs, i.e., upsets in memory flags, that are in addition filtered through threshold gates (type (1) guards use local timeouts and are thus considered non-faulty, and all type (2) guards employed by the main state machine and the quick cycle use thresholds). Note that due to the logical masking of metastability provided by the threshold gates (cf. Section 6.2.3) any memory flag acts as an implicit synchronizer: If a faulty node successfully induces metastability in the flag, this does not matter until the threshold can actually be reached. If the respective time span is large, the memory flag is likely to have stabilized again already. Therefore, in addition to succeeding in creating metastability, faulty nodes must do so within a specific window of time. Due to the asynchronously triggered transitions, this window of vulnerability of the synchronizing stage for *Syn* is very small. The resulting very low probability of a metastable upset due to a fault is considered acceptable.

The residual probability of metastable upsets could be further reduced by introducing synchronizer stages. Considering their performance penalty of one extra clock cycle on the one hand and the low initial risk of metastable upsets (that are handled by the system level fault tolerance with much lower average performance penalty) on the other hand, however, the introduction of synchronizers does not seem beneficial in general.

##### Correctness.

Thanks to the synchronous TSM described above, the maximum state transition time $d_{\mathrm{Trans}}$ can easily be expressed in terms of the frequency of the pausable clock. Hence, it is reasonably easy to see that the HSM satisfies the specification given in Section 4, when it starts from a proper initial state and avoids the above scenarios (a) and (b) of unstable guards. A careful simulation analysis of the overall HSM design confirms that it can in fact recover from arbitrary initial states, except metastable ones. With respect to metastable initial states, we conjecture that eventual recovery occurs with probability 1 due to the fact that the only devices used in the implementation that are not forgetful are flip-flops with dominant reset (in the TSM sequence counter) and Muller C-gates (in the control logic of the ASM), for both of which it is known that metastability eventually resolves.

### 6.4 Clocks and watchdog timers

#### 6.4.1 Pausable oscillator

The TSM clock is an asynchronously startable and synchronously stoppable ring oscillator, which provides a clock signal *TSMClock* that is 0 when the clock is stopped via an active 1 input signal *TSMCStop*. A variant that is also asynchronously stoppable (under certain timing constraints) is used for driving the watchdog timers (see below). The frequency of the ring oscillator is primarily determined by the (odd) number of inverters in the feedback loop.^25^ It varies heavily with the operating conditions, in particular with supply voltage and temperature: The resulting (two-sided) clock drift *ξ* is typically in the range of 7 to 9% for uncompensated ring oscillators like ours; in ASICs, it could be lowered down of 1 to 2% by special compensation techniques [15]. Note that the two-sided clock drifts map to ϑ=(1+ξ)/(1−ξ) bounds roughly between 1.15 and 1.19 or 1.02 and 1.04, respectively.

##### Correctness.

**Fig. 13:**
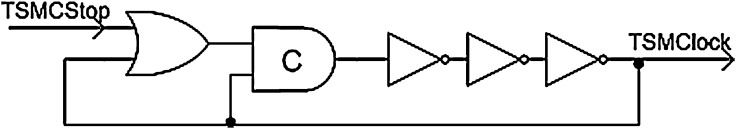
Pausable ring oscillator implementing the TSM clock.

The operation of the TSM clock circuit shown in Fig. 13 is straightforward: In its initial state, *TSMCStop* = 1 and the Muller C-gate has 1 at its output, so *TSMClock* = 0. Note that the circuit also stabilizes to the initial state if the Muller C-gate was erroneously initialized to 0, as the ring oscillator would eventually generate *TSMClock* = 1, enforcing the correct initial value 1 of the C-gate. When the ASM requests a state transition, at some arbitrary time when a transition guard becomes true, it just sets *TSMCStop* = LOW. This starts the TSM clock and produces the first rising edge of *TSMClock* half a clock cycle time later. As long as *TSMCStop* remains 0, the ring oscillator runs freely.

The stopping of *TSMClock* is regularly initiated by the TSM itself: With the rising edge of *TSMClock* that moves the TSM into *Trm*, *TSMCStop* is set to 1. Since *TSMClock* is also 1 after the rising edge,^26^ the output of the C-gate is forced to 1 as well. Hence, after having finished the half period of this final clock cycle, the feedback loop is frozen and *TSMClock* remains 0.

##### Metastability.

The problem of devising a proof that the pausable clock will eventually recover when it starts from a metastable initial state is intricate (and outside the scope of this paper); this is not obvious due to the quite complex feedback loop involved in this circuit. We conjecture that similar arguments as in [44] can be used to show that this will happen with probability 1; with this result established one could hope to infer that the HSM as a whole recovers from arbitrary metastable states with probability 1.

For metastability-free operation of the C-gate in Fig. 13, (a) the falling transition of *TSMCStop* must not occur simultaneously with a rising edge of *TSMClock*, and (b) the rising transition of *TSMCStop* must not occur simultaneously with the falling edge of *TSMClock*. (a) is guaranteed by stopping the clock in state *Trm* of the TSM, since the output of the C-gate is permanently forced to 1 on this occasion; *TSMClock* cannot hence generate a rising transition before *TSMCStop* goes to 0 again. Whereas this synchronous stopping normally also ensures (b), we cannot always rule out the possibility of getting *TSMCStop* = 1 close to the *first* rising edge of *TSMClock*: (b) could thus occur due to prematurely disabled type (3) guards, which we discussed already with respect to their potential to create metastability in the TSM, recall Section 6.3. Besides being a rare event, this can only do harm during stabilization, however.

#### 6.4.2 Watchdog timer design

Every ASM state, except for *accept* in Fig. 8, is associated with at most one watchdog timer required for type (1) guards; *accept* is associated with three timers (for $T_{1}$ and $T_{2}$ as well as for $T_{2}^{+}$ in Fig. 6). Recall that a timer is reset by the TSM when its associated state is entered, which does not necessarily happen synchronously with its counting clock.

**Fig. 14:**
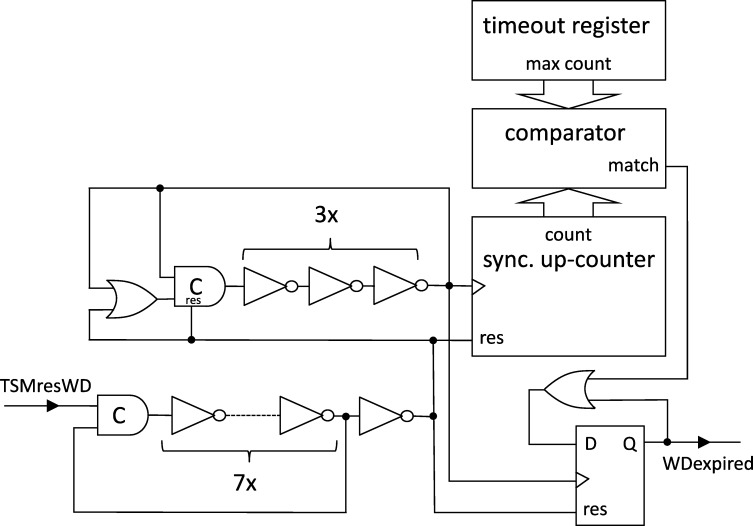
Implementation principle of a watchdog timer.

According to Fig. 14, every watchdog timer consists of a synchronous, dominantly resettable up-counter that is clocked by its own pausable oscillator (as shown in Fig. 13) and a timeout register that holds the timeout value TO.^27^ A comparator raises an output signal if the counter value is equal to the TO register value. A “capture flip-flop” with dominant reset memorizes the *expired* condition until the timer is re-triggered. Note that using a (synchronous) flip-flop instead of an SR latch here allows us to completely mask glitches at the comparator output, which may originate from intermediate inconsistent bit patterns at the counter output.

The reset signal *TSMresWD*, supplied by the TSM, (re-)triggers the watchdog as follows: The counter is reset to zero, the capture flip-flop is cleared, and the oscillator is temporarily stopped. Stopping the oscillator is necessary to avoid metastability effects due to the unsynchronized release of the reset signal (recall that this signal originates from the clock domain of the TSM!) and the watchdog's local oscillator. Note carefully, however, that the Muller C-gate in Fig. 13 must be extended by a dominant reset input connected to its stop input (*TSMCStop*) to prevent metastable upsets. Moreover, to ensure a proper reset, one has to make sure that the reset duration is sufficiently large. To guarantee this, *TSMresWD* is fed into a pulse shaping circuitry (bottom left part of Fig. 14) that makes sure that the reset pulse is longer than one period of the local clock.^28^ At the end of this shaped reset pulse, counter and flip-flop have attained a clean reset state, and the local oscillator has safely been brought to a stable stopped state (with its output at 0). When reset is finally released (to 0), the oscillator starts running. As soon as the comparator detects a match between the current count and the timeout register, it will set match to 1. This rising edge is captured by the flip-flop, thus keeping the watchdog timeout signal *WDexpired* at 1 even when the comparator reverts its output to 0 later on again (note that the counter keeps on running). This construction ensures that the oscillator continues to operate also after the timeout expires, which is crucial for self-stabilization; in a system where the clocks driving the timeouts can be permanently halted, there is no way to avoid deadlocks for all possible states.

As for the watchdog timer with random timeout $R_{3}$ in Fig. 10, our implementation uses a *linear feedback shift register* (LFSR) that is continuously clocked by the watchdog's oscillator: A uniformly distributed random value from the specified range, sampled from the LFSR, is loaded into the timeout register whenever the watchdog timer is re-triggered.^29^ If both the watchdog timer and the LFSR are clocked by the same oscillator, this can be done in a synchronous way. In order to avoid metastable upsets of the LFSR, which might occur when stopping the clock upon retriggering the watchdog as described above, we use a standard pausable oscillator for $R_{3}$: Since $R_{3}$ is guaranteed to timeout before it is re-triggered (see Fig. 10), we can stop the oscillator synchronously (as in the TSM) when the timeout occurs, i.e., tie its stop input to the OR of *WDexpired* and the pulse-shaped reset signal. Another add-on is needed for the random timer $R_{3}$ in order to guarantee that the LFSR recovers from an arbitrary state after a fault: Since an LFSR has a forbidden internal state (all-0 in our case), we use an additional comparator that detects an all-0 LFSR output and forces a (synchronized) reset of the LFSR to a proper initial state.

##### Metastability.

Using a dominant reset in conjunction with a reset pulse of sufficient length guarantees that pausable oscillator, counter, and flip-flop cannot become metastable when a watchdog timer is re-triggered. Since all other activities are driven by the local oscillator and hence trivially metastability-free, this leaves the pulse-shaping unit as the only component that could possibly suffer from a metastable upset. However, *TSMresWD* is generated by the TSM, which is guaranteed to generate a clean pulse at every correct node. Hence, the pulse shaping unit could become metastable only at a faulty node. With respect to the recovery from a metastable state, similar considerations as for the memory flags in Section 6.2.2 suggest that the pulse shaping unit will stabilize to an initial state with reset set to 0 eventually with probability 1.

##### Correctness.

Combining the implementations of the pausable oscillator (with additional reset) and the watchdog timer, it is not too difficult to verify that the specification given in Section 4 is met, provided all circuits start from a non-metastable initial state.

With respect to self-stabilization, the pulse shaping unit can be guaranteed to stabilize to an initial state with its reset output 0 from an arbitrary internal state. Hence, the pausable oscillator and hence the counter will eventually run. Provided that the counter implementation guarantees that it cycles through the full (finite) sequence of possible states (unless reset earlier), i.e., there are no deadlock states or alternative cyclic sequences that might be entered in case of a fault, our implementation ensures that *WDexpired* will eventually be set to 1, even if started from an arbitrary initial state. One should bear in mind, though, that the time to recover a watchdog timer contributes to the overall stabilization time of the system. It is hence advisable to make sure that recovering a watchdog timer does not take much longer than the largest timeout value in the system, e.g. by avoiding oversized counter registers.

### 6.5 Computing the end-to-end delay bounds

From the implementations of the individual components, it is straightforward to compute the delays *d*, $d_{\min}^{+}$, and $d_{\max}^{+}$. Recall that *d* bounds, for any node *i*, the maximal time that passes between a state transition of a remote node and a possibly triggered corresponding state change, i.e., the transition of $S_{i}$. This is done by computing the maximal sum of delays of any possible computing path, ranging over all possible state transitions (cf. Fig. 5), taking into account the delay of the channels $S_{j,i}$. Clearly, the channel delay for the remote channels $S_{j,i}$ exceeds the delays of the local channels; hence, the longest path to the input ports of the state transition module is bounded by $d_{\mathrm{Chan}}+d_{\mathrm{Mem}}+d_{\mathrm{Th}}>d_{\mathrm{Time}}$. Subsequently, the HSM locks the state transition and the TSM executes, which takes about two and a half clock cycles *C* of the pausable oscillator. Note, however, that the new state is written into the flip-flops holding the state already during the *commit* cycle, i.e., after at most 1.5*C*. A more accurate bound on *d* than $d_{\mathrm{Chan}}+d_{\mathrm{Mem}}+d_{\mathrm{Th}}+2.5C$ is thus

$$d⩽1.5C+\max\{C,d_{\mathrm{Chan}}+d_{\mathrm{Mem}}+d_{\mathrm{Th}}\}.$$

For our approach, $d_{\max}^{+}\approx d$, since the only difference to *d* is that $d_{\mathrm{Chan}}$ is replaced by $d_{\mathrm{Chan}}^{+}$, the delay of the simpler 1-bit channels (cf. Section 6.2.1). If the main state machine's channels would utilize serial encoding, though, one might well have that $d_{\mathrm{Chan}}\gg \max\{d_{\mathrm{Chan}}^{+},C\}$. Finally, $d_{\min}^{+}>C/\vartheta$, since this is the minimal time the HSM allows between locking a state transition and actually performing the transition at the port $S_{i}$.^30^

## 7 Experimental evaluation

Our prototype implementation has been written in VHDL and compiled for an Altera Cyclone IV FPGA using the Quartus tool, see [47].

Since FPGAs neither natively provide the basic elements required for asynchronous designs nor allow the designer to exercise control over the actual mapping of functions to the available LUTs (we implemented threshold modules via LUTs rather than via combinational AND-OR networks for complexity reasons), we had to make sure that properties that hold naturally in “real” asynchronous implementations also hold here. Apart from standard functional and timing verification via Modelsim, we therefore conducted some preliminary experiments for verifying the assumed properties (glitch-freeness, monotonicity, etc.) of the synthesized implementations of our core building blocks. Backed up by the (positive) results of these experiments, complete systems consisting of n=4 respectively n=8 nodes (tolerating at most f=1 respectively f=2 Byzantine faulty nodes) have been built and verified to work as expected; overall, they consume 23000 respectively 55000 logic blocks. Note however, that both designs also include the test environment, which makes up a significant part of the setup.

To facilitate systematic experiments, we developed a custom test bench that provides the following functionality:

(1) Measurement of pulse frequency and skew at different nodes.
(2) Continuous monitoring of the potential for generating metastability in HSM state transitions.
(3) Starting the entire system from an arbitrary state (including memory flags and timers), either specified deterministically or chosen at random.
(4) Resetting a single node to some initial state, at arbitrary times.
(5) Varying the clock frequency of any oscillator, at arbitrary times.
(6) Choosing the communication delay between each pair of sender and receiver.

All these experiments can be performed with Byzantine nodes. To this end, the HSMs of the Byzantine nodes can be replaced by special devices that allow to (possibly inconsistently) communicate, via the communication data buses, any HSM state to any receiver HSM at any time. Points (1) to (6) are achieved as follows:

(1) is accomplished using standard measurement equipment (logic analyzer, oscilloscope, frequency counter) attached to the appropriate signals routed via output pins.

(2) is implemented by memorizing any event where more than one guard is enabled (at the time when the TSM locks a state transition) in a flag that can be externally monitored.

(3) is realized by adding a scan-chain to the FPGA implementation, which allows us to serially shift-in arbitrary initial system states at run-time. Repeated random experiments are controlled via a Python script executed at a PC workstation, which is connected via USB to an ATMega 16 microcontroller (μC) that acts as a scan-controller towards the FPGA. For each experiment, the Python script generates a bit-stream representing an initial configuration. The μC takes this stream, sends it to the FPGA via the serial scan-chain interface, and finally signals the FPGA to start execution of FATAL^+^. Simultaneously, it starts a timer. When a timeout occurs or the FPGA signals completion of the experiment, the μC informs the Python script which records the time until completion together with the outcome of the experiment and proceeds with sending the next initial configuration.

To enable (4) to (6), the testbench provides a global high-resolution clock that can be used for triggering mode changes. To ensure its synchrony w.r.t. the various nodes' clocks, we replaced all start/stoppable ring oscillators by start/stoppable oscillators that derive their output from the global clock signal. Point (4) is achieved by just forcing a node to reset to its initial state for this run at any time during the current execution. In order to facilitate (5), dividers combined with clock multipliers (PLLs) are used: For any oscillator, it is possible to choose one of five different frequencies (0, excessively slow, slow, fast, excessively fast) at any time. For (6), a variable delay line implemented as a synchronous shift register of length X∈[0,15], driven by the global clock, can be inserted in any data bus connecting different HSMs individually.

In order to exercise also complex test scenarios in a reproducible way, a dedicated *testbed execution state machine* (TESM), driven by the global clock, is used to control the times and nodes when and where clock speeds, transmission delays, and communicated fault states are changed and when a single node is reset throughout an execution of the system. Transition guards may involve global time and any combinatorial expression of signals used in the implementation of FATAL^+^, i.e., any predicate on the current system state.

**Fig. 15:**
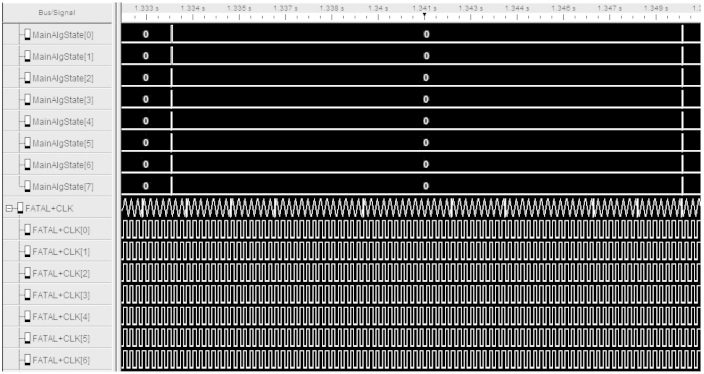
FATAL and FATAL^+^ clocks: MainAlgState[i]=1 iff *i* is in *accept*, and FATAL + CLK[i] is *i*'s FATAL+ signal.

Using our testbench, it was not too difficult to get our FATAL^+^ implementation up and running. With the implementation parameters ϑ=1.3, d=13T, $d_{\min}^{+}=d_{\max}^{+}=3T$, where T=400ns (2.5 MHz) is the experimental clock period, and minimal timeouts according to the constraints listed in [13] (cf. Section 5.7), pulses of an 8 node FATAL respectively FATAL^+^ system (including the quick cycle) occur at a frequency of about 62 Hz respectively 10 kHz. A logic analyzer screenshot is depicted in Fig. 15. Note that the quite low values for the frequency stem from the fact that we were intentionally slowing down the system, enabling better control of the execution.

As to be expected from such a fairly complex setup, we spotted several hidden design errors that showed up during our experiments, but also some minor, yet problematic errors in our theoretical analysis (like a missing factor of *ϑ* in one of our timeouts due to a typo). In the original setup, these issues manifested in deviations of the measured w.r.t. the predicted performance. After resolving them, we conducted the following experiments, observing the behavior of both the overall FATAL^+^ and the underlying FATAL pulse generation protocol.

### 7.1 Worst-case skew experiment

To drive an 8-node FATAL system into a worst-case skew scenario,^31^ the set of nodes was split into four sets:

• A set *A* of two nodes with slow clock sources. All communication to these nodes is maximally delayed.
• A set *B* of two nodes with fast clock sources. All communication to these nodes is minimally delayed.
• Another set *C* of two nodes with fast clock sources. All communication to these nodes is maximally delayed.
• A set *D* of two faulty nodes. These nodes always send *propose* to the nodes in *B* and do not send any other signals.^32^

**Fig. 16:**
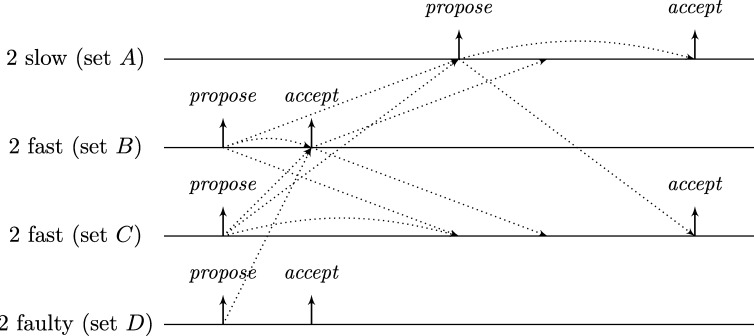
Worst-case skew scenario for a FATAL system of *n*=8 nodes with *f*=2 faulty nodes.

This setup leads to the following behavior of the main state machine (Fig. 8) once the system is stabilized. The nodes in B∪C will always switch to *propose* first because their timeouts $T_{3}$ expire (it is shown in [13] that at this time ${\text{next}}_{i}=1$ at all non-faulty nodes), and due to the “help” of the faulty nodes, the threshold of n−f=6 for switching to *accept* is reached at the nodes in *B* after the minimal delay. It takes the maximal delay until the nodes in *A* realize that 4⩾f+1 nodes reached state *propose* and switch to this state. Since 4<n−f, the nodes in A∪C require the support of the nodes in *A* to follow to state *accept*. Hence, this happens another maximal delay later. The resulting scenario is depicted in Fig. 16. Assuming that the communication delay is at most *d* and at least $d_{\min}⩾0$, we predict a skew of at most $2d-d_{\min}$ between the nodes in *B* switching to *accept* and the nodes in A∪C catching up.

**Fig. 17:**
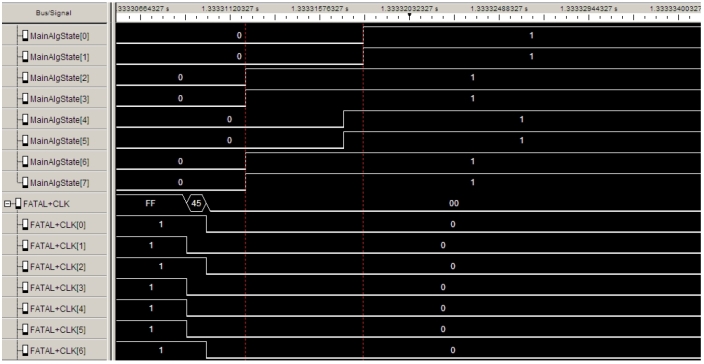
Logic analyzer screenshot from worst-case skew experiment for a FATAL system with *f*=2 faulty nodes (6 and 7): MainAlgState[i]=1 iff *i* is in *accept*, and FATAL + CLK[i] is *i*'s FATAL+ signal. The nodes in *C* (4 and 5) switch to *accept* earlier than those in *A* (0 and 1) because the clock driving their TSM is faster.

The experimental results confirmed the analytic predictions as being essentially tight: Letting the fast nodes run at a speed of 3 MHz and the slow nodes at 2.5 MHz, and setting the maximum delay *d* to about 3.6 μs (9 clock cycles), we observed a skew of about 6 μs. This is consistent with the relatively large minimum delay $d_{\min}$ arising in our testbed. A logic analyzer screenshot is depicted in Fig. 17.

The figure also demonstrates the capability of FATAL^+^ to generate pulses with significantly less skew (1 μs) on top of the FATAL pulses with worst-case skew.

### 7.2 Metastability experiments

We run a series of experiments dedicated to finding situations that potentially lead to metastable upsets. We repeatedly set up 8-node systems with randomly chosen clock speeds between 2.5 MHz and 3.25 MHz and communication delays of at most 16 clock cycles. While the system stabilized from these random initial states, we monitored the nodes' HSM state transitions after stabilization for multiple active conflicting state transitions during a period of over 60 h in total. As predicted by our theoretical findings, in none of the experiments two conflicting guards were ever active at the same (global testbench) time after stabilization.

### 7.3 Stabilization time experiments

We evaluated stabilization time both in the absence and in the presence of faulty nodes. In the latter case, we demonstrated the influence of the choice of the random timeout $R_{3}$ on the stabilization time.

#### 7.3.1 Stabilization in the absence of faulty nodes

**Fig. 18:**
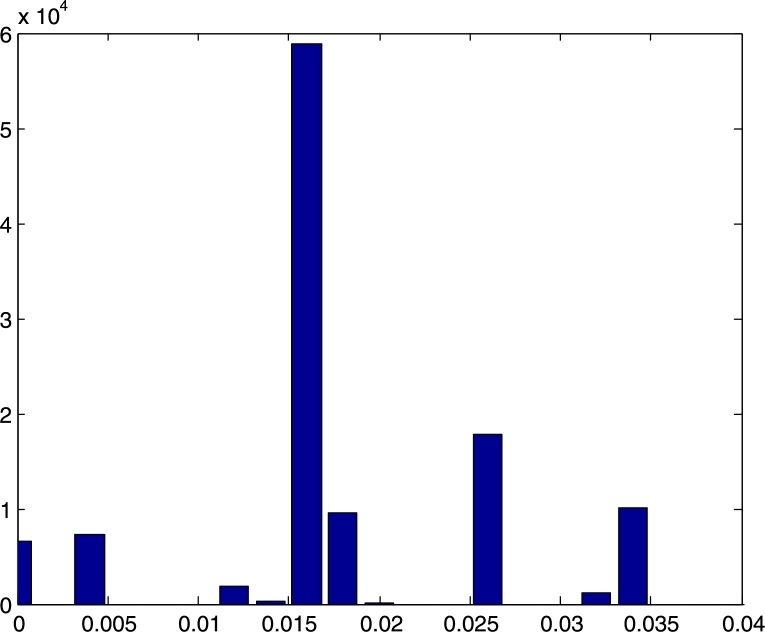
Head of distribution of stabilization times (in s) for over 250 000 randomly initialized 8-node instances.

**Fig. 19:**
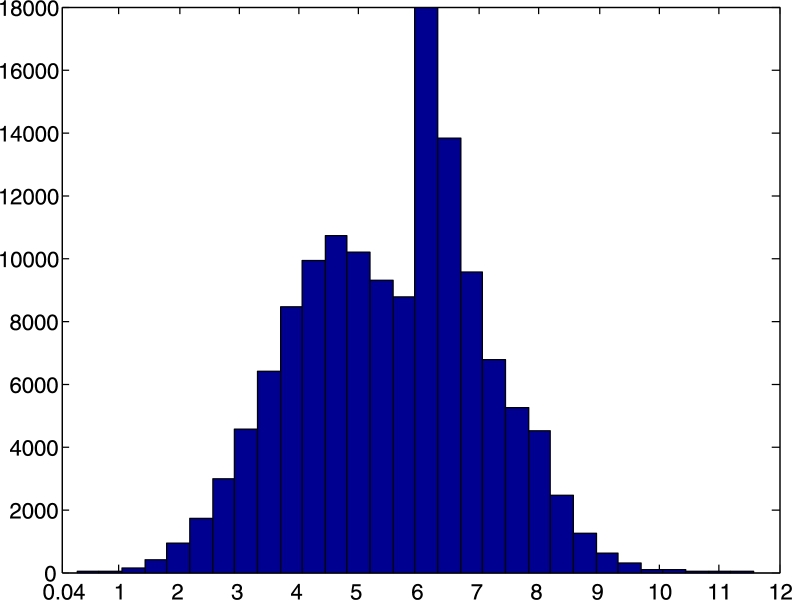
Tail of distribution of stabilization times (in s) for over 250 000 randomly initialized 8-node instances.

To evaluate stabilization times in the absence of faulty nodes, we set up an 8 node system and run over 250000 experiments in each of which the nodes booted from random initial states, with randomly chosen clock speeds between 2.5 MHz and 3.25MHz=2.5ϑMHz, and message delays of up to d=16 clock cycles. As soon as all nodes switched to state *accept* within 2*d* time, the FPGA signaled the μC to record the elapsed time and start the next experiment. A considerable fraction of the scenarios (over 45%) stabilizes within less than 0.035 s (less than 5500*d*), which can be credited to the fast stabilization mechanism intended for individual nodes resynchronizing to a running system (see Fig. 18). The remaining runs (see Fig. 19; please mind the different *y*-axis scale) stabilize, supported by the resynchronization routine, in less than 12 s (about $1.9\cdot 10^{6}d$), which is less than the system's upper bound on $R_{3}$ of approximately 14.9 s (about $2.3\cdot 10^{6}d$) and significantly less than the system's upper bound on $T_{\mathrm{slow}}$ given in Theorem 5.2, which is no more than 44.5 s (about $7\cdot 10^{6}d$) in this scenario. Note that the stabilization time is inversely proportional to the frequency, i.e., in a system that is not artificially slowed down, stabilization is orders of magnitude faster. For example, assuming d=1ns, we obtain that over 45% of the experiments stabilize within 5.5 μs, and all experiments stabilize within 1.9 ms.

Experimental results carried out for a 4-node system were analogous.

**Fig. 20:**
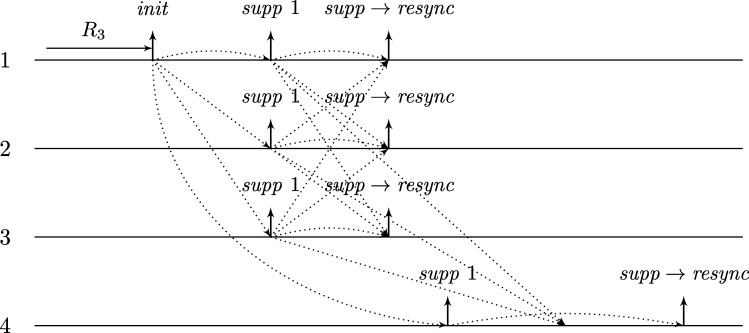
Synchronization by the Resynchronization Algorithm with 4 nodes in the absence of faulty nodes.

Either the main algorithm was capable to stabilize by itself (as for a large fraction the experiments in the head of the distribution), or once the resynchronization algorithm provided support after $R_{3}$ expired at some node and n−f nodes switched to *resync* in approximate synchrony (the experiments in the tail of the distribution). Fig. 20 shows stabilization by the resynchronization algorithm in a 4-node system: Eventually, all nodes switch to state *none*. A node whose timeout $R_{3}$ expires at a time when all timeouts $\left(R_{2},\mathrm{supp}\right)$ are expired, say node 1, forces all nodes from *none* into supp1. Additional $R_{3}$ timers, expiring at other nodes, may only force nodes into *supp* *j*, with j≠1, but do not prevent nodes from eventually communicating *supp* to all other nodes. Thus nodes finally switch to supp→resync and from there to *resync*, in synchrony. This again suffices to deterministically stabilize the nodes' main algorithm (as shown in [13]). Note that the condition that all corresponding $R_{2}$ timeouts are expired when a timeout $R_{3}$ expires (actually, n−f suffice) will eventually be satisfied. This happens at the latest when $R_{3}$ expires for the second time at some node, simply because the distribution of the randomized timeout $R_{3}$ guarantees that the picked duration is always larger than (roughly) $\vartheta R_{2}$.

#### 7.3.2 Stabilization with Byzantine nodes and deterministic timeouts

**Fig. 21:**
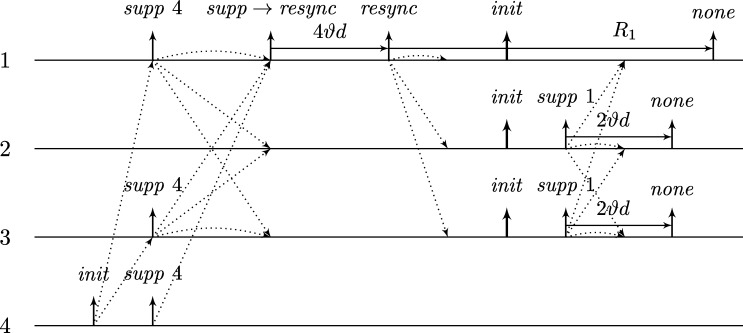
Execution of the Resynchronization Algorithm with 4 nodes and equal timeouts in presence of a Byzantine faulty node (node 4).

**Fig. 22:**
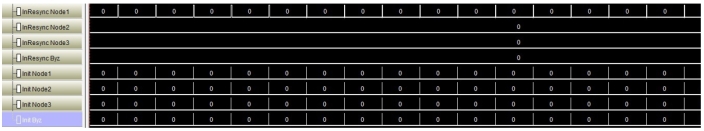
Logic analyzer screenshot of an execution of the Resynchronization Algorithm of a 4 node FATAL system with equal $R_{3}$ timeouts and a Byzantine node: InResync Node*i*=1 iff *i* is in *resync*, and Init Node*i*=1 iff *i* is in *init*.

The importance of timeout $R_{3}$ being randomly distributed is demonstrated in the following experiment. We set up a 4-node FATAL+ system with one Byzantine faulty node, say node 4, and chose all $R_{3}$ to be equal and initially synchronized, i.e., all $R_{3}$ timeouts expire at about the same time at all correct nodes. If the Byzantine node knows when $R_{3}$ is going to expire, it can prohibit correct nodes from simultaneously switching to *resync*, thereby preventing synchronization of the Main Algorithm and thus stabilization: Shortly before $R_{3}$ expires at the correct nodes, it sends *init* to two nodes, say 1 and 3, making them switch to supp4. Subsequently, however, it only supports node 1 by sending *supp* to it. This forces node 1 to switch to supp→resync and then *resync* alone. While node 1 is in *resync* (i.e., while $R_{1}$ is running), it does not support other nodes by sending *supp*. Specifically, it does not support nodes 2 and 3 when they switch to supp1. Eventually all nodes switch back to *none*, and the scenario can be repeated. Fig. 21 depicts the scenario and Fig. 22 shows a logic analyzer screenshot of this experiment. Note, however, that by definition of the probability distribution of $R_{3}$, executions where $R_{3}$ expires in synchrony at all correct nodes forever occur with probability 0.

We remark that there is always a nonzero probability that the randomly chosen durations of the timeouts $R_{3}$ at non-faulty nodes align in a fortunate manner, so that stabilization could not be prevented even by an omniscient adversary orchestrating clock drifts, message delays, and faulty nodes. While the probability of such a convenient event occurring in O(n) time decreases exponentially in the number of nodes *n*, it is reasonably likely for n=7 and in particular n=4 (i.e., systems that tolerate f=2 or f=1 faults, respectively). This observation has been verified for n=4 by the first of the two experiments below.

#### 7.3.3 Stabilization with Byzantine nodes and probabilistic timeouts

Two experimental setups were chosen to test stabilization in the presence of Byzantine nodes, using probabilistic timeouts $R_{3}$ for correct nodes. In the first experiment, a Byzantine node has access to the timeout values of all nodes as soon as they start their $R_{3}$ timers. In this case, the Byzantine node followed the strategy from before, obstructing any stabilization attempt that would otherwise be successful. We observed that the Byzantine node was able to block at most one stabilization attempt of each non-faulty node. Then it failed to prevent stabilization because the $R_{2}$ timeouts corresponding to the Byzantine node did not expire on time before some non-faulty node successfully initialized stabilization.

In the second experiment, the Byzantine node has no access to timeout values, and therefore simply sends inconsistent *init* and *supp* signals as often as allowed by the timeouts $R_{2}$ corresponding to it. We did not observe any inhibited synchronized switch to *resync* when $R_{3}$ expired at a correct node, however.

It should be noted that weaker adversaries and “better” initial configurations result in a constant stabilization time, irrespectively of the number of nodes *n* (see [13] for details). The second experiment above demonstrates such a case; Theorem 5.3 states another. The common-case stabilization time will therefore be considerably smaller than the (probabilistic) worst-case bound that is linear in *n*.

## 8 Conclusions

In this work, we introduced a novel modeling framework for self-stabilizing, fault-tolerant asynchronous digital circuits and demonstrated its applicability to our recently introduced FATAL^+^ clock generation scheme for multi-synchronous GALS architectures. Our framework enables to reason about high-level properties of the system based on the behavior of basic building blocks, at arbitrary granularity and in a seamless manner. At the same time, the hierarchical structure of the model permits to do this in a fashion amenable to formal analysis. We believe this to be the first approach concurrently providing all these features, and therefore consider it as a promising foundation for future research in the area of fault-tolerant digital circuits.

As the conclusion of our paper, we now assess to which extent the properties of our implementation of the FATAL^+^ algorithm, which have been expressed and verified within our modeling framework and tested experimentally, meet our design goals. Furthermore, we will discuss a number of potential improvements and future research avenues. Our exposition will follow the optimization criteria listed in Section 2.1.7.

• **Area consumption:** For a suitable implementation, the total number of gates is O(nlogn) per node. This can be seen by observing that the complexity of the threshold gates is dominating the asymptotic number of gates, since the O(n) remaining components of a node have a constant number of gates each; using sorting networks to implement threshold gates, the stated complexity bound follows [48]. Trivially, this number of gates is a factor of O(logn) from optimal. We conjecture that in fact this complexity is asymptotically optimal, unless one is willing to sacrifice other desirable properties of the algorithm (e.g. optimal resilience). Assuming that the gate complexity of the nodes adequately represents the area consumption of our algorithm, we conclude that our solution is satisfactory in that regard.
• **Communication complexity:** Our implementation uses 7 (1-bit) wires per channel, and sequential encoding of the states of the main state machine would reduce this number to 5. All communication are broadcasts. Considering the complexity of the task, there seems to be very limited room for improvement.
• **Stabilization time:** Our algorithm has a stabilization time of O(n) in the worst case. Recent findings [49] show that a polylogarithmic stabilization time can be achieved at a low communication complexity; however, this comes at the expense of suboptimal resilience, a weaker adversarial model, and, most importantly, constants in the complexity bounds that make the resulting algorithm inferior to our solution for any practical range of parameters. Moreover, as formalized in [13] and demonstrated in Section 7, for a wide range of scenarios our algorithm achieves constant stabilization time. Considering the severe fault model, we conclude that despite not being optimal, our algorithm performs satisfactory with respect to this quality measure.
• **Resilience:** It is known that 3f+1 nodes are necessary to tolerate *f* faults [25,14] unless cryptographic tools are available. Since the complexity incurred by cryptographic tools is prohibitive in our setting, our algorithm features optimal resilience.
• **Delays:** As mentioned, the delay of wires is outside our control. Taking $d_{\min}^{+}$ and $d_{\max}^{+}$ into account in the quick cycle machine, we make best use of the available bounds in terms of the final frequency/synchrony trade-off. The delays incurred by the computations performed at nodes are proportional to the depths of the involved circuits. Again, the implementation of the threshold gates is the dominant cost factor here. The sorting network by Ajtai et al. [48] exhibits depth O(logn). Assuming constant fan-in of gates, this is clearly asymptotically optimal if the decision when to increase the logical clock $L_{v}$ next indeed depends on all n−1 input signals of *v* from remote nodes. We conclude that, so far as within our control, the design goal of minimizing the incurred delays is met by our algorithm.
• **Metastability:** We discussed several effective measures to prevent metastability in Section 6. Our experiments support our theoretical finding that, after stabilization, metastability may not occur in absence of further faults. However, since metastability is an elusive problem for which it is difficult to transfer insights and observations to other modes of operation of a given system—let alone to different implementation technology—a mathematical treatment of metastability is highly desirable. Our model opens up various possible approaches to this issue. For one, it is feasible to switch to a more accurate description of signals in terms of signals' voltages as continuous functions of time. Another option choosing an intermediate level of complexity would be to add an additional signal state (e.g. ⊥) for “invalid” signals, representing e.g. creeping or oscillating signals. Assigning appropriate probabilities of metastability propagation and decay to modules, this would enable a unified probabilistic analysis of metastability generation, propagation, and decay within a modeling framework using discrete state representations. Such an approach could yield entirely unconditional guarantees on system recovery; in contrast, our current description requires an *a priori* guarantee that metastability is sufficiently contained during the stabilization process.
• **Connectivity:** The algorithm presented in this work requires to connect all pairs of nodes and is therefore not scalable. Unfortunately, it is known that $\Omega (n^{2})$ links are required for tolerating f∈Ω(n) faults in the worst case [26,27]. We argued for the assumption of worst-case behavior of faulty nodes; however, it appears reasonable that typical systems will not exhibit a worst-case *distribution* of faults within the system. Indeed, many interesting scenarios justify to assume a much more benign distribution of faults. In the extreme case where faults are distributed uniformly and independently at random with a constant probability, say, 10%, of a node being faulty, node degrees of Δ∈O(clogn) would suffice to guarantee (at a given point in time) that the probability that more than Δ/9 neighbors of any node are faulty, is at most $1-1/n^{c}$. Note that this implies that the mean time until this property is violated polynomially *grows* with system size. Using the FATAL^+^ protocol in small subsystems (of less than Δ nodes), system-wide synchronization will be much easier to achieve than if one would start from scratch. In this setting, Δ∈O(logn) would replace *n* in all complexity bounds of the FATAL^+^ algorithm, resulting in particular in gate complexity O(lognloglogn) per node, computational delay O(loglogn), and stabilization time O(clogn) with probability $1-1/n^{c}$. Thus, this approach promises “local” fault-tolerance of Ω(Δ) faults in each neighborhood in combination with excellent scalability in all complexity measures, and realizing this is a major goal of our future work.
• **Clock size:** The constraint (1) entails that either clock size is bounded or large clocks result in larger stabilization time. This restriction can be overcome if we use the clocks of bounded size generated by FATAL^+^ as input to another layer that runs a synchronous consensus algorithm in order to agree on exponentially larger clocks [41].

Finally, we would like to mention two more prospective extensions of our work. First, building on our modeling framework, it seems feasible to tackle an even more strict verification of the algorithm's properties than “standard” mathematical analysis. The hierarchical structure and formal specifications of modules seem amenable to formal verification methods. Such an approach should benefit from the possibilities to adjust the granularity of the model by the distinction between basic and compound modules as well as the restrictions imposed by the module specifications; more restrictive modules may be simpler to analyze, yet will guarantee the same properties as the stated variants. Second, it should be noted that it is straightforward to derive clocks of even higher frequency from the FATAL^+^ clocks. This is essentially done by frequency multiplication, at the expense of increasing the clock skew. We refer to Dolev et al. [13] for details.

Overall, we consider the present work an important step towards a practical, ultra-robust clocking scheme for SoC. We plan to address the open problems discussed above in the future, and hope that this will ultimately lead to dependable real-world systems clocked by variants of the scheme proposed in this article.

## Footnotes

3 Or, to be scientifically accurate, we rather successfully failed at falsifying them. Our implementation primarily serves as a proof of concept, as clearly an FPGA implementation can merely hint at the properties of an ASIC.

4 We use real-valued, unbounded clocks here to simplify the presentation. It will later become clear that the algorithm can indeed operate with discrete bounded clocks, as it does not need to access absolute clock values, but rather approximately measures bounded differences in time.

5 This is the reason why we speak of an abstract channel. We will later introduce the (physical) channels that essentially represent the wires on the chip; the maximal delay *d* is then the sum of the maximal delay of the physical channels and the computing elements of the nodes.

6 The only restriction is that transient faults do not affect the non-volatile memory (and in particular not the algorithm itself), as this would induce a permanent fault.

7 Allowing cryptography would still necessitate n>2f[26,27]; we hence discard this option due to the additional complexity incurred.

8 We are aware that this constitutes a serious scalability issue; again we refer to the discussion in Section 8.

9 One could compromise by guaranteeing that nodes recover in bounded time, provided that the number of faults is never overwhelming. In fact, the algorithm presented in this article has the property that in this case nodes will recover faster and deterministically (in contrast to the slower, probabilistic stabilization from arbitrary system states). However, sacrificing stabilization from arbitrary states will not reduce the complexity of the algorithm significantly, and theory strongly indicates that the respective gain is limited to a constant factor in general.

10 Note that in this regard our approach is superior to standard GALS systems using synchronizers, where the risk of metastability is immanent (at every clock transition) also in normal operation.

11 It is worth mentioning, though, that the asymptotic increase in the number of events per time unit that could cause metastable upsets is clearly at least polynomial in *n*. As synchronizer chains decrease the probability of upsets exponentially, the required length of synchronizer chains will asymptotically grow as Ω(logn), increasing the system cost and (effectively) decreasing its operational frequency.

12 If a single event such as e.g. an ionizing particle hit can render multiple nodes faulty, even tolerating a large number of faulty nodes is of little use w.r.t. the overall resilience of the system.

13 In other words, the module must recover from arbitrary corruptions of its memory.

14 In practice, one would of course need an input conveying the control signal starting the oscillator; we conveniently hide this detail within the module specification.

15 Note that if any submodule is already covered by $E_{0}$, it must not have any output port and due to non-emptiness the only possible (trivial) execution on the output ports is always feasible.

16 We remark that, technically speaking, excessively high voltages on the input wires could also be interpreted as an “input violation”, as this violates the definition of our signals. However, it makes sense to interpret such (hopefully exceptional) events as a fault of the module.

17 Recall that during stabilization we cannot exclude metastability with certainty even in the absence of any further faults.

18 It is not self-evident that this type of masking is very effective for metastability as well. Later on we will discuss why this is indeed the case.

19 To match our model, invalid signal states are simply mapped to some default state, e.g. *resync* for the main state machine.

20 It is, however, possible to replace parallel communication by serial communication, e.g., by extending the (synchronous) TSM (see Section 6.3) appropriately.

21 Note that there is some freedom with respect to the mapping of module ports to the physical system, which also affects which module(s) become(s) infeasible due to a (physical) fault. However, no matter what the precise mapping, care has to be taken to avoid correlated failures. For instance, if all channels meet in a single spot due to bad routing, manufacturing defects or electromigration could connect several channels, therefore rendering our system-level fault-tolerance (i.e., the resilience to *f* node failures) ineffective.

22 Our basic modules appear simple enough to be amenable to formal verification. Still, there are complications: Besides the fact that we assume not only continuous time but also continuous computations, which rules out using standard verification approaches, there is the challenge of finding and expressing suitable input port execution constraints required for implementation correctness. Exploring this avenue is part of our future work.

23 Formally, this abstraction builds upon a weakened definition of lower-level channels, which attain values from $S\cup \left(\begin{array}{c} S\\ 2 \end{array}\right)$. Alternatively, it would also be possible to use an explicit transition state ⊥ (encoded by any bit sequence not corresponding to a state), and force the sender to always perform state transition via ⊥.

24 Some dedicated experiments confirmed that even our LUT implementation on an FPGA, for which we have no control over the placement, operates glitch-free on monotonic inputs.

25 In our FPGA implementation, the oscillator frequency is so high that, to reduce the hardware overhead for this proof-of-concept implementation, we also employ a frequency divider at the output.

26 Obviously, we only have to take care in the timing analysis that setting *TSMCStop* = 1 occurs well within the first half period.

27 Note that these values must be hard-coded in order to avoid that a fault that intuitively should be transient (e.g. a bit flip in volatile memory) becomes permanent by “altering the algorithm”.

28 This is why seven inverters are shown in Fig. 14, indicating that the output pulse needs to remain active for more than two half periods of the local clock, assuming three inverters in its oscillator's loop.

29 Note that for many settings, it is reasonable to assume that the new random value remains a secret until the timeout expires, as it is not read or in any other way considered by the node until then. Under this condition, FATAL^+^ is resilient against the strong adversary specified in [13]. As our prototype implementation is not meant for studying security issues, however, the simple LFSR implementation is sufficient here.

30 Clearly, a more precise analysis would yield tighter bounds.

31 The maximal imprecision is meaningful in connection with the system's frequency only. In contrast, the skew captures the maximal time difference between corresponding signal transitions at the nodes, which reflects the quality of synchronization without requiring additional context.

32 In our model, this behavior is mapped to a default signal at the receivers, e.g. *resync* in the main state machine.
